# FAT4 overexpression promotes antitumor immunity by regulating the β-catenin/STT3/PD-L1 axis in cervical cancer

**DOI:** 10.1186/s13046-023-02758-2

**Published:** 2023-09-01

**Authors:** Dongying Wang, Shuying Wu, Jiaxing He, Luguo Sun, Hongming Zhu, Yuxuan Zhang, Shanshan Liu, Xuefeng Duan, Yanhong Wang, Tianmin Xu

**Affiliations:** 1 Obstetrics and Gynecology Department, The Second Hospital of Jilin University, 218 Zi Qiang Street, Changchun, Jilin 130041 China; 2 National Engineering Laboratory for Druggable Gene and Protein Screening, Northeast Normal University, Changchun, Jilin 130024 China; 3 Cancer Institute, The Affiliated Hospital of Qingdao University, Qingdao University, Qingdao, 266071 China

**Keywords:** FAT4, Wnt/β-catenin pathway, PD-L1, CTL, Cervical cancer

## Abstract

**Background:**

FAT4 (FAT Atypical Cadherin 4) is a member of the cadherin-associated protein family, which has been shown to function as a tumor suppressor by inhibiting proliferation and metastasis. The Wnt/β-catenin pathway activation is highly associated with PD-L1-associated tumor immune escape. Here, we report the mechanism by which FAT4 overexpression regulates anti-tumor immunity in cervical cancer by inhibiting PD-L1 N-glycosylation and cell membrane localization in a β-catenin-dependent manner.

**Methods:**

FAT4 expression was first detected in cervical cancer tissues and cell lines. Cell proliferation, clone formation, and immunofluorescence were used to determine the tumor suppressive impact of FAT4 overexpression in vitro, and the findings were confirmed in immunodeficient and immunocomplete mice xenografts. Through functional and mechanistic experiments in vivo and in vitro, we investigated how FAT4 overexpression affects the antitumor immunity via the β-catenin/STT3/PD-L1 axis.

**Results:**

FAT4 is downregulated in cervical cancer tissues and cell lines. We determined that FAT4 binds to β-catenin and antagonizes its nuclear localization, promotes phosphorylation and degradation of β-catenin by the degradation complexes (AXIN1, APC, GSK3β, CK1). FAT4 overexpression decreases programmed death-ligand 1 (PD-L1) mRNA expression at the transcriptional level, and causes aberrant glycosylation of PD-L1 via STT3A at the post-translational modifications (PTMs) level, leading to its endoplasmic reticulum (ER) accumulation and polyubiquitination-dependent degradation. We found that FAT4 overexpression promotes aberrant PD-L1 glycosylation and degradation in a β-catenin-dependent manner, thereby increasing cytotoxic T lymphocyte (CTL) activity in immunoreactive mouse models.

**Conclusions:**

These findings address the basis of Wnt/β-catenin pathway activation in cervical cancer and provide combination immunotherapy options for targeting the FAT4/β-catenin/STT3/PD-L1 axis.

**Graphical Abstract:**

Schematic cartoons showing the antitumor immunity mechanism of FAT4. (left) when Wnts bind to their receptors, which are made up of Frizzled proteins and LRP5/6, the cytoplasmic protein DVL is activated, inducing the aggregation of degradation complexes (AXIN, GSK3β, CK1, APC) to the receptor. Subsequently, stable β-catenin translocates into the nucleus and binds to TCF/LEF and TCF7L2 transcription factors, leading to target genes transcription. The catalytically active subunit of oligosaccharyltransferase, STT3A, enhances PD-L1 glycosylation, and N-glycosylated PD-L1 translocates to the cell membrane via the ER-to-Golgi pathway, resulting in immune evasion. (Right) FAT4 exerts antitumor immunity mainly through following mechanisms: (i) FAT4 binds to β-catenin and antagonizes its nuclear localization, promotes phosphorylation and degradation of β-catenin by the degradation complexes (AXIN1, APC, GSK3β, CK1); (ii) FAT4 inhibits PD-L1 and STT3A transcription in a β-catenin-dependent manner and induces aberrant PD-L1 glycosylation and ubiquitination-dependent degradation; (iii) Promotes activation of cytotoxic T lymphocytes (CTL) and infiltration into the tumor microenvironment.
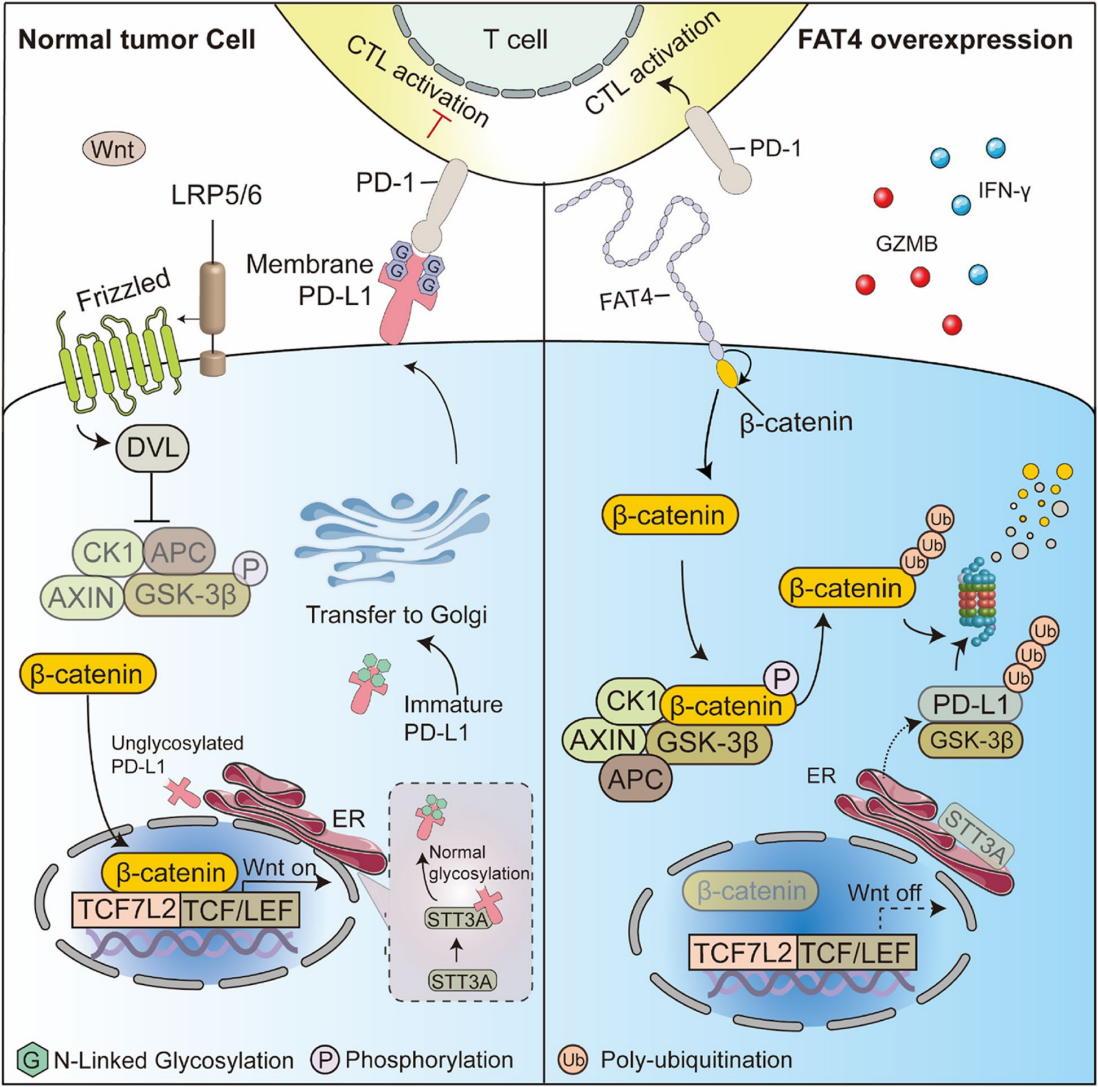

**Supplementary Information:**

The online version contains supplementary material available at 10.1186/s13046-023-02758-2.

## Introduction

The FAT cadherin family, which includes FAT1, FAT2, FAT3, and FAT4, is a large atypical calmodulin superfamily in mammals [1]. *Fat4* knockout mice die at birth and exhibit defective PCP in tissues including the kidney, inner ear, neural tube [2], and cerebral cortex [3]. FAT4 is the closest vertebrate homolog of *Drosophila* Fat (ft) and plays a role in controlling planar cell polarity (PCP) [4] and regulating the Hippo signaling [5]. FAT4 protein is located to the cell membrane in mammalian cells and is thought to have tumor-suppressive effects; double allele FAT4 inactivation promotes tumorigenicity in mouse mammary epithelial cell lines [6]; however, its role in tumor immunity is unknown.

Aberrant activation of the Wnt/β-catenin pathway drives the progression of a variety of human malignancies, including cervical cancer [7, 8]. Cadherin-related proteins interact with β-catenin in normal epithelial cells and sequester it in the cell periphery, regulating epithelial cell development and intracellular adhesion [9, 10]. In the Wnt/β-catenin pathway, β-catenin is a key effector controlled by Wnt ligands and regulates gene transcription. Wnt ligands activate cytoplasmic protein disheveled (DVL), which inhibits degradation complexes (AXIN, GSK3, CK1, and APC). Non-phospho-β-catenin then translocates into the nucleus and interacts with T cell-specific factor (TCF)/lymphoid enhancer-binding factor (LEF) transcription factors to activate Wnt target genes [11, 12]. Notably, β-catenin has recently been identified as a transcription factor for the *CD274* and *STT3* gene that promotes the N-glycosylation and stabilization of PD-L1 [13, 14]. The tumor microenvironment (TME) is a source of both standard and non-standard Wnt ligands that can induce aberrant activation of Wnt signaling in cancer cells, leading to epithelial-mesenchymal transition (EMT) and altered immune responses [15, 16]. Furthermore, β-catenin activation modulates regulatory T cells (Treg) survival as well as tumor cell escape from immune surveillance [13, 17]. Since the Wnt/β-catenin pathway is essential for cancer cell survival and immune editing, it is a profitable target for antitumor immunity.

Immune checkpoint blockade (ICB) therapies have shown to be effective in metastatic melanoma and non-small cell lung cancer [18, 19], and clinical trials in cervical cancer and other cancers are currently underway [20], however drug resistance and recurrence remain common [21]. The programmed cell death 1 (PD-1) receptor is found on activated T cells, and its ligand PD-L1, is a major co-inhibitory checkpoint signal used by tumors to block the cytotoxic T lymphocyte (CTL) activity, promote T cell apoptosis, and allow cancer cells to escape immune surveillance [22]. Recent research has discovered that tumor PD-L1 expression levels can be used to predict clinical response to anti-PD-L1/PD-1 therapy [23]. The extracellular domain of PD-L1 has four N-X-T/S motifs (N35, N192, N200, and N219), all of which can be N-glycosylated [24]. N-glycosylation occurs mainly in the endoplasmic reticulum (ER) and Golgi apparatus, where glycosyltransferases transfer N-acetylglucosamine to the N-X-T/S motif in the ER lumen, followed by PD-L1 transport to the Golgi apparatus for further maturation and folding by glycosidases, which translocate to the cell membrane to promote immune escape [25, 26]. Importantly, the oligosaccharyltransferase complex (OST) catalytic subunit STT3 is required for PD-L1 glycosylation and protein stabilization, while β-catenin binding to the transcription factor TCF7L2 is essential for the induction of STT3A/B transcription[14]. Glycosylation stabilizes PD-L1 and protects PD-L1 from GSK3β-mediated 26S proteasome-dependent ubiquitination degradation [24]. In general, glycosylation and ubiquitination modifications of PD-L1 can cross-regulate each other and promote PD-L1 stability. As a result, expanding our understanding of the regulation of PD-L1 expression is critical for developing anti-PD-L1/PD-1 therapy and promoting cancer immunotherapy [27].

In this study, we discovered that FAT4 inhibited Wnt/β-catenin signaling by antagonizing nuclear localization of β-catenin and promoting phosphorylation and degradation of β-catenin by the “destruction degradation complex (AXIN1, APC, GSK3, CK1)”. Furthermore, FAT4 inhibits PD-L1 and STT3A transcription in a β-catenin-dependent manner and induces aberrant PD-L1 glycosylation; specifically, FAT4 overexpression inhibited PD-L1 ER-Golgi transport and cell membrane localization, attenuated STT3A-PD-L1 interaction, and thus induced polyubiquitination-dependent degradation of PD-L1. Importantly, FAT4 overexpression was able to promote complete tumor regression in C57BL/6 mouse models of cervical cancer, and we collected tumors prior to tumor regression and found that FAT4 overexpression promoted cytotoxic T lymphocyte (CTL) infiltration and activation. Our findings suggest that FAT4 enhances antitumor immune responses by inhibiting the β-catenin/STT3/PD-L1 signaling pathway and that FAT4 is promising as a new target for combination immunotherapy.

## Methods

### Clinical specimens

All immunohistochemical specimens, including 50 pairs of cervical squamous cell carcinoma and paracancerous tissues and 20 pairs of normal cervical specimens, were obtained from patients who underwent surgical resection in the Second Hospital of Jilin University from 2013 to 2017, and all patients did not receive radiotherapy or chemotherapy before operation. In addition, for immunoblotting, we collected 12 pairs of fresh surgically removed cervical squamous cell carcinoma and paraneoplastic tissues. The study was authorized by the Institute Research Ethics Committee (No.2020-146) before written consent from all patients.

### Bioinformatic analysis

Datasets involved were downloaded from the TCGA (The Cancer Genome Atlas) Dataset of CESC (Cervical squamous cell carcinoma and endocervical adenocarcinoma).

### Cell culture

Human cervical cancer cell lines Hela, Caski, SiHa, C33A, and ME180 were obtained from the Chinese Academy of Sciences Shanghai Institute for Biological Sciences Cell Resource Center. Mouse cervical cancer cell line U14 was obtained from the National Infrastructure of Cell Line Resource (Beijing). Hela, Caski, SiHa cells were cultured in Roswell Park Memorial Institute (RPMI) 1640 medium (SH30255.FS, Hyclone, Logan, UT, USA), C33A and U14 cells were cultured in Dulbecco modified essential medium (DMEM, SH30285. FS, Hyclone), while ME180 cells were cultured in McCoy’s 5A medium (SH30270.01, Hyclone). All medium was supplemented with 10% fetal bovine serum (FBS, FB15015, Clark), 100 U/ml penicillin, and streptomycin antibiotics (P1400, Solarbio, Beijing, China) at 37℃ in 5% CO_2_. All cell lines have been tested for mycoplasma contamination and were validated by short tandem repeat (STR) polymorphism analysis performed by the Genetica DNA Laboratories.

### Vectors

The deficient Cas9-synergistic activation mediator (dCas9-SAM) system was used to endogenously enhance *FAT4/Fat4* mRNA expression. The dCas9-VP64-Puro was used to express wild-type dCas9, and sgRNA-MS2-P65-HSF1-Neo was used to target the FAT4/Fat4 promoter region. For sgRNA sequences: sg*FAT4* #H1: GGCTGTAGGCGGTCTGGTGT; #H2: TAGCATCCCGAGAAGCCAGT; sg*Fat4* #M1: GATTATGCAGCTGACTGCCA; sg*Fat4* #M2: GTGGAAGAGAACATTGGAGA.

### Establishment of FAT4 overexpressing cell lines

The day before lentivirus transduction, the specified cell lines were grown in 6-well plates at a density of 5 × 10^4^ cells/well (20–30% density). The media containing the dCas9-VP64-Puro lentivirus (MOI = 10) was cultured for another 12 h. 48 h later, the appropriate concentration of puromycin was added to the cell cultures to obtain a mixed clonal cell line stably expressing dCAS9-VP64. And then one or more sgRNA-MS2-P65-HSF1 lentiviruses were infected, G418 screened, and western blotting and qPCR were performed to identify the overexpressed clones.

### Mice

This study was approved and reviewed by the Institutional Animal Care and Use Committee (IACUC) of Jilin University (KT202102006). Mice used in all experiments were obtained from Charles River, and the animals’ health and immune status were normal. Animal facilities that house mice are regularly checked for standard pathogens, and mice are housed on a 12/12-h light/dark cycle with a maximum of 5 per cage, with free access to food and water. For the immunodeficient mouse models, sg*FAT4* human cervical cancer ME180 cells, sg*Fat4* mouse cervical cancer U14 cells, and their CTRL groups (1 × 10^6^ cells suspended in 50 µl PBS, 1:1 mixed with Matrigel Matrix) were injected into the left dorsal region of 6-week-old female BALB/c nude mice (Charles River).

For the immune-competent mouse models, sg*Fat4* mouse cervical cancer U14 cells and CTRL group (1 × 10^6^ cells suspended in 50 µl PBS, 1:1 mixed with Matrigel Matrix) were injected subcutaneously into the left dorsal region of 6-week-old female C57BL/6 female mice (Charles River). Tumor diameters were measured and recorded every 3 days. Tumor volume (mm^3^) was calculated by measuring the longest diameter and shortest diameter of the tumor: Volume = (shortest diameter)^2^ × longest diameter × 0.5. After 2 weeks, 5 mice were randomly selected for euthanasia, and tumors were collected, weighed, and processed to prepare frozen sections, flow cytometry, and paraffin-embedded tissue. The remaining mice were used to monitor and record survival to 60–90 days. Animals were euthanized in advance when the tumor growth exceeded 10% of the original body weight of the animal, the average tumor diameter exceeded 20 mm (not more than 2000m^3^), or the tumor metastasized or rapidly grew to ulceration, resulting in infection or necrosis and other adverse indicators.

### FACS analysis of CTL profiles

All flow cytometry antibodies used in this study were listed in Supplementary Table 1. U14 xenograft tumors were exfoliated quickly and gently, and single-cell suspensions were produced after physical grinding and filtering. After blocking with Anti-Mouse CD16/CD32 (553140, BD Biosciences) antibody, the cells were incubated in a medium containing PMA/Ionomycin mixture (1x) and BFA/Monensin Mixture (1x) for 5 h at 37 °C and protected from light. Cells were stained with CD45-Brilliant Violet 510™ (30-F11), CD3-PE (17A2), CD4-FITC (GK1.5), CD8-FITC (53-6.7), PD-1-APC (29F.1A12) in FACS Buffer. After fixation and permeabilization by Foxp3/Transcription Factor Staining Buffer Set (00-5521-00, eBioscience, Thermo Scientific), intracellular GZMB and IFN-γ were stained using IFN-γ-PECY7 (4S.B3), Granzyme B-APC (QA16A02) antibody. Stained cells were analyzed by BD FACSCanto II (BD Biosciences) cytometer. Data were processed by the FlowJo 10.8 software.

### Immunofluorescence

Fresh mouse tumor mass was isolated and embedded in OCT blocks frozen to prepare 6 mm thick frozen sections. Cells were inoculated on coverslips at the bottom of a 48-well plate (2–4 × 10^4^ cells/well). Cells and tissues were fixed in 4% paraformaldehyde for 30 min at room temperature (RT) and then permeabilized in 0.5% Triton X-100 solution for 10 min. Non-specific sites were blocked with 5% bovine serum albumin for 30 min at RT and incubated with primary antibody overnight at 4 °C. After washing with PBS, CoraLite488 or 594 secondary antibodies (Proteintech) were incubated for 1 h at RT, then stained with Hoechst (Solarbio) for 10 min at RT. Coverslips with the anti-fading solution were mounted on glass slides and observed under a Zeiss LSM 880 confocal microscope.

### Protein extraction, western blot, and co-immunoprecipitation

Cells or tissues are lysed in lysis buffer and the supernatant fractions are collected. SDS-PAGE is used to separate proteins, which are then transferred to polyvinylidene difluoride (PVDF) membranes. The PVDF membranes were blocked in 5% skimmed milk for 1 h at RT before being treated with specified primary antibodies overnight at 4 °C. The membranes were then incubated with horseradish peroxidase‐conjugated secondary antibodies. For immunoprecipitations, the specified antibody was precleared with protein A/G beads (MedChemExpress, Shanghai, China) for 2 h and then rotational incubated with cell lysates overnight at 4 °C. The protein A/G beads were rinsed four times with phosphate-buffered saline containing Tween 20 after incubation (PBST), then the samples were analyzed by immunoblotting. Enhanced chemiluminescence was used to detect immunoreactive bands. Supplementary Table 1 has detailed antibody information.

### T cell-mediated tumor cell killing assay

After euthanasia of mice, spleens were ground in a 70 μm cell sieve, and lymphocytes suspended in culture medium were collected. After centrifugation, erythrocyte lysate (R1010, Solarbio) was added and resuspension. The extracted mouse lymphocytes were cultured in Dynabeads^®^ Mouse T-Activator CD3/CD28 (11452D; Life Technologies, Thermo Scientific) and Recombinant Mouse IL-2 (1000 U/mL, HZ-1015, Proteintech) for one week according to the manufacturer’s protocol. After allowing the cancer cells to attach to the culture dish overnight, they were treated with activated T cells for 48 h (1:3). Cells and cell debris were removed by PBS washing, and then live cancer cells were quantified by spectrophotometry at OD (570 nm), followed by crystalline violet staining.

### PD-1 and PD-L1 binding assay

Cells were fixed in 4% paraformaldehyde for 30 min at RT and then incubated with recombinant human PD-1 Fc protein (0.1 µg/mL, 1086-PD, R&D Systems) or recombinant mouse PD-1 Fc protein (1 µg/mL, 1021-PD, R&D Systems) for 1 h. After washing with PBS, cells were incubated with Alexa Fluor 488-labeled Goat anti-Human/Mouse IgG (H + L) Cross-Adsorbed Secondary Antibody (A-11013, A-31561; Life Technologies, Thermo Scientific) for 1 h at RT, followed by staining with Hoechst (C0030, Solarbio) for 10 min at RT. Alexa Fluor 488 fluorescence intensity was measured using a microplate reader Synergy Neo (BioTeK, Winooski, VT, USA), and cells were visualized using a confocal microscope (LSM880, Carl Zeiss). Five different microscope images were randomly selected for quantitative analysis.

### Immunohistochemistry

All of the specimens were formalin-fixed and embedded in paraffin wax. For IHC assays, paraffin-embedded tissues are dewaxed and antigens are repaired at 115 °C for 20 min using a Tris-EDTA (PH = 9.0) based unmasking solution. After blocking endogenous peroxidase activity with 3% hydrogen peroxide, the non-specific antigen was blocked using 10% goat serum for 30 min at room temperature, followed by incubation of the sections with primary antibody at 4 °C overnight. After washing with PBS for 3 × 5 min, slices were treated with a Histostain-Streptavidin-Peroxidase kit (SP9001, ZSZB-Bio), followed by 3,3′-diaminobenzidine (DAB) staining and hematoxylin counterstaining. All tissue slices were photographed using a DP72 microscope (Olympus Corporation, Tokyo, Japan). A double-blind procedure was used to assess the sections. Two pathologists used a semi-quantitative scoring system to examine the sections. The number of positive cells was scored as 4 (> 75%), 3 (51–75%), 2 (25–50%), 1 (5–25%), or 0 (5%), while the staining intensity was assessed as 3 (brown), 2 (light brown), 1 (light yellow), or 0 (colorless). The immunoreactivity score (IRS) was calculated by adding the two grades. Negative (0), weak (1–3); moderate (4 and 5); and strong (6 and 7) were used to categorize the total score. For each group, the IRS median value was computed.

### Kaplan-Meier survival analysis

Cervical cancer cases were divided into two groups based on the immunohistochemical score mentioned above: high expression (moderate and strong) and low expression (negative and weak). Progression-free survival was calculated for follow-up data. In the first 2 years, the follow-up period was 3 months. In the next 3 to 5 years, the follow-up period was 6 months, and in the subsequent years, the follow-up period was 12 months. The survival period was described using the Kaplan-Meier curve, and the log-rank test was used to compare the survival periods of each group. *P* value < 0.05 was considered statistically significant (*P* < 0.05).

### RNA isolation and qRT-PCR

Total RNA Extraction Kit (R1200, Solarbio) was used to extract total RNAs, which was then reverse transcribed into cDNA using the TransScript^®^ One-Step gDNA Removal and cDNA Synthesis SuperMix (TransGen Biotech) according to the manufacturer’s instructions. FastStartTM SYBR Green Master (Roche Diagnostics) was used to perform RT-qPCR according to the manufacturer’s protocol. The sequence information for each primer used for human gene expression analysis was as follows:

- FAT4-Forward: 5′- CAAATGCTGTGATTGCGTAT-3′,
- FAT4-Reverse: 5′- AACAGTGGCAAAGCTACACCT-3′
- STT3A-Forward: 5′- GAAGCAACAGGATTCCACCTACC-3′,
- STT3A- Reverse: 5′- CAATGGACGGAGAAGAGTAGGC-3′
- CD274-Forward: 5′- TGGCATTTGCTGAACGCATTT-3′,
- CD274-Reverse: 5′- TGCAGCCAGGTCTAATTGTTTT-3′
- ACTB-Forward: 5′- CGTGCGTGACATTAAGGAGAAG-3’,
- ACTB-Reverse: 5′- GGAAGGAAGGCTGGAAGAGTG-3’.

The sequence information for each primer used for mouse gene expression analysis was as follows:

- Fat4-Forward: 5′-GAGCCAATCCTTCAGAGGAGG-3′,
- Fat4-Reverse: 5′-CCATGACCAGAAGTCCACACA-3′
- Stt3a-Forward: 5′-ACCATCGTTACGTACCACCT-3′,
- Stt3a-Reverse: 5′-AGCCAGCTACAGATCGAGAA-3′
- Cd274-Forward: 5′-AATCGTGGTCCCCAAGCCTC-3′,
- Cd274-Reverse: 5′-ACAGCAGGCTGTGAATATAATGC-3′
- Actb-Forward: 5′-GATCCTGACCGAGCGTG-3′,
- Actb-Reverse: 5′-GTTGGCATAGAGGTCTTTACGG-3′.

For quantification of gene expression, the 2-^ΔΔ^Ct method was used. β-actin expression was used for normalization.

### Cell proliferation and colony formation

For CCK-8 cell proliferation assays, cells (1000 per well) were cultured in 96-well plates and cell proliferation was detected using a cell counting kit-8 (96992, Sigma-Aldrich) at 450 nm for 3 days. In the colony formation assay, 500 cells were inoculated in 6-well plates with 3 replicate wells per group. 7 d later, cell colonies were stained with Giemsa (G1015, Solarbio) and colonies of more than 50 cells were counted under the microscope.

### Edu proliferation assay

pEGFP-N1/PD-L1 was a gift from Mien-Chie Hung (Addgene plasmid # 121478) [24]. CTRL and sgRNA-*FAT4* ME180 cells were transfected with plasmid DNA using X-tremeGENE™ HP DNA Transfection Reagent (Roche, 06366236001). Click-iT^®^ c5-ethynyl-20-deoxyuridine (EdU) kit (C10337, Invitrogen) was used to detect the cell proliferation ability, and the cells were seeded in confocal plates at a density of 5 × 10^5^ cells per well, and mixed with Incubate with 50 μM EdU buffer. After 2 h at 37 °C, fix with 4% formaldehyde for 0.5 h, permeabilize with 0.1% Triton X-100 for 20 min, add Click-iT^®^ reaction mixture, and stain Hoechst cell nuclei. Fluorescence microscopy was used to visualize the results.

### Plasmid transfection and luciferase activity assay

pLV-β-catenin deltaN90 was a gift from Bob Weinberg (Addgene plasmid # 36985) [28]. M50 Super 8 × TOPFlash (Addgene plasmid # 12456) and M51 Super 8 × FOPFlash (Addgene plasmid # 12457) were gifts from Randall Moon [29]. ME180 cells were transfected with plasmid DNA using X-tremeGENE™ HP DNA Transfection Reagent (Roche, 06366236001) for 24 h. Cells were lysed and subjected to luciferase reporter assay by using a Double-Luciferase Reporter Assay Kit (TransGen Biotech, Beijing, China) and was normalized to pSV40-Renilla luciferase activity.

### Statistical analysis

GraphPad Prism 8.0 software was used for statistical analysis (GraphPad Software, Inc. La Jolla, CA). In all graphs, data points and bars represent the means of independent biological replicates, error bars represent standard deviations, and all data are reported as mean ± standard deviation (SD). All data represent similar results from at least three independent experiments. Data were analyzed for significance using Student’s t-test (two groups) or one-way ANOVA with Tukey’s post hoc test (multiple groups), correlations were analyzed using Pearson’s correlation coefficient, and data on clinicopathological characteristics of CC patients were analyzed using the χ2 test. *P* < 0.05 was considered a statistically significant difference.

## Results

### FAT4 is downregulated in cervical cancer tissues and cervical cancer cells

**Fig. 1:**
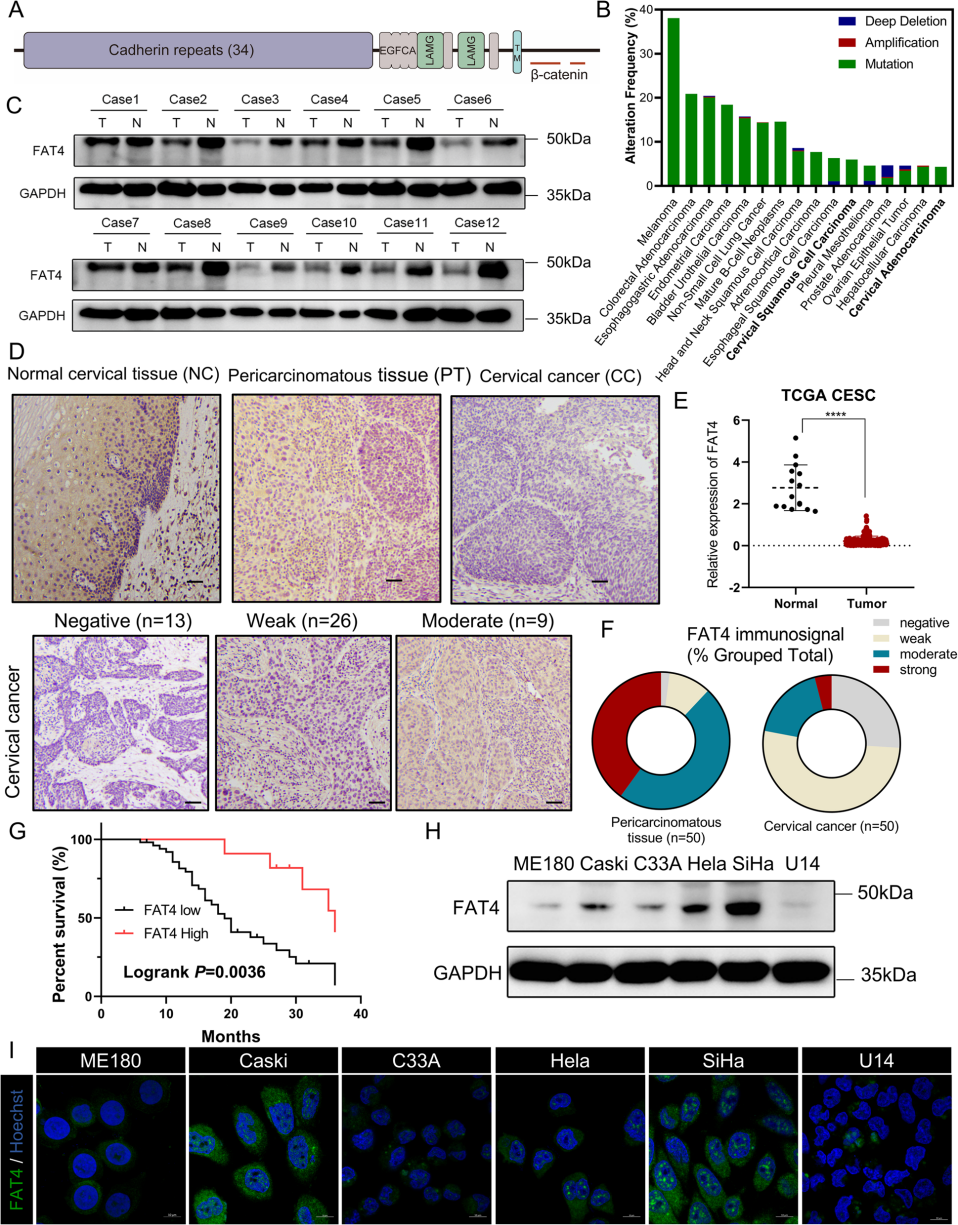
FAT4 downregulated in cervical cancer and is associated with poor prognosis. **A** Schematic diagram of the functional structural domains of FAT4 protein. Red lines indicate possible β-catenin binding regions. (TM, transmembrane domain; LAMG: laminin G-like domain; EGFCA: EGF-like repeat). **B** Using the cBioPortal online analysis tools, data from the TCGA database were extracted and analyzed for cross-cancer *FAT4* gene alterations. The frequency of *FAT4* gene mutation, amplification, and profound deletion across cancers was shown by histograms. The results showed that *FAT4* is frequently mutated or deleted in numerous types of human cancer.** C** Western blot showing FAT4 protein levels in 12 pairs of pericarcinomatous tissues and cervical cancers, GAPDH was loaded as a control. **D** Representative IHC images showing FAT4 expression in normal cervical (NC) tissues, pericarcinomatous tissue (PT), and cervical cancer (CC) tissues. Note that in normal cervical tissue, FAT4 is highly expressed in the cytomembrane of stratum superficiale and stratum spinosum cells (inserts show ×2 magnification). Scale bar = 100 μm. **E** The FAT4 mRNA expression data were obtained from TCGA and compared with normal tissues. **F** Immunoreactivity score of pericarcinomatous tissue and cervical cancer tissues. **G** Kaplan-Meier survival curves in 50 cervical cancer patients based on high (red) or low (black) FAT4 immunological scoring (*P* = 0.0036). **H**&**I** FAT4 protein expression was detected by (H) immunoblotting and (I) immunofluorescence in ME180, Caski, C33A, Hela, SiHa and U14 cervical cancer cell lines. FAT4 protein expression was relatively low in human-derived C33A and ME180 cell lines, and murine-derived U14 cell lines. Note that even in the cell lines with high FAT4 expression (SiHa, Hela, and Caski), the cell membrane localization of FAT4 was not evident, nuclei stained with Hoechst (blue)

The Human *FAT4* gene encoded a 4924-aa protein with extracellular 34 Cadherin repeats, 4 EGF-like domains, and 2 Laminin G-like domains (Fig. 1A) [1]. The TCGA database was analyzed using the cBioPortal online tools [30] and *FAT4* is frequently mutated or deleted in human tumors, especially squamous cell carcinomas, implying that *FAT4* dysregulation is a prevalent alteration in human solid tumors. Further analysis of the Cervical squamous cell carcinoma and endocervical adenocarcinoma (CESC) dataset revealed that FAT4 mRNA levels were significantly lower in tumor samples than in normal tissues (Fig. 1B). FAT4 expression was subsequently examined in twelve groups of human cervical cancer tissues and cervical pericarcinomatous tissues (PT), revealing that FAT4 protein levels in cervical cancer tissues were significantly lower than in pericarcinomatous tissues (Fig. 1C). To further confirm these results, we performed immunohistochemical analysis on 50 cervical cancer patients and 20 normal cervical specimens. In both normal squamous epithelium tissues and pericarcinomatous tissues, the strong FAT4 immunosignal was found in the cytoplasm and cytomembrane (Fig. 1D), and FAT4 is low expression in cervical cancer tissues (78% versus 22%, Fig. 1F). The expression of FAT4 was divided into two groups based on the immunohistochemical score (IRS): low expression (IRS ≤ 3) and high expression (IRS ≥ 4). According to the Kaplan-Meier survival analysis, cervical cancer patients with low FAT4 expression have a poor prognosis (Logrank *P* = 0.0036, Fig. 1G). The relationship between FAT4 expression and clinical histopathological characteristics was also examined in this work, low FAT4 expression was thought to be related to aggressive and metastatic characteristics, including cervical invasion depth, lymphovascular invasion, and local lymph node metastasis (Table1).

**Table 1:** Relationship between FAT4 expression and clinicopathological characteristics in cervical cancer

	Samples	**FAT4 expression**			
		Low	High	χ2	*P*-value^a^
**All patients**	50	39	11		
**Age distribution**				1.237	0.2660
Median age < 60	34	25	9		
Median age ≥ 60	16	14	2		
**Tumor size (cm)**				0.2617	0.6089
< 4	38	29	9		
≥ 4	12	10	2		
**HPV-DNA**				2.658	0.1030
Positive	43	36	7		
Negative	7	3	4		
**TCT**				0.5036	0.4779
Positive (≥ ASC-US)	39	32	7		
Negative	11	7	4		
**FIGO 2018 stage**				2.131	0.1443
IA1- IB2	36	30	6		
IB3-IIA1	14	9	5		
**Cervical invasion**				10.28	0.0013**
1/2	20	11	9		
≥ 1/2	30	28	2		
**Lymphovascularinvasion**				7.219	0.0072**
Yes	19	11	8		
No	31	28	3		
**PLN metastasis**				5.003	0.0253*
Yes	19	18	1		
No	31	21	10		
**Endometrial/uterine corpus metastasis**				3.096	0.0785
Yes	9	9	0		
No	41	30	11		

*ASC-US* Atypical squamous cells of undetermined significance

^*^P < 0.05, **P < 0.01

^a^χ2 text; Significant different

### FAT4 overexpression suppresses proliferation in cervical cancer cells and immunodeficient mice

**Fig. 2:**
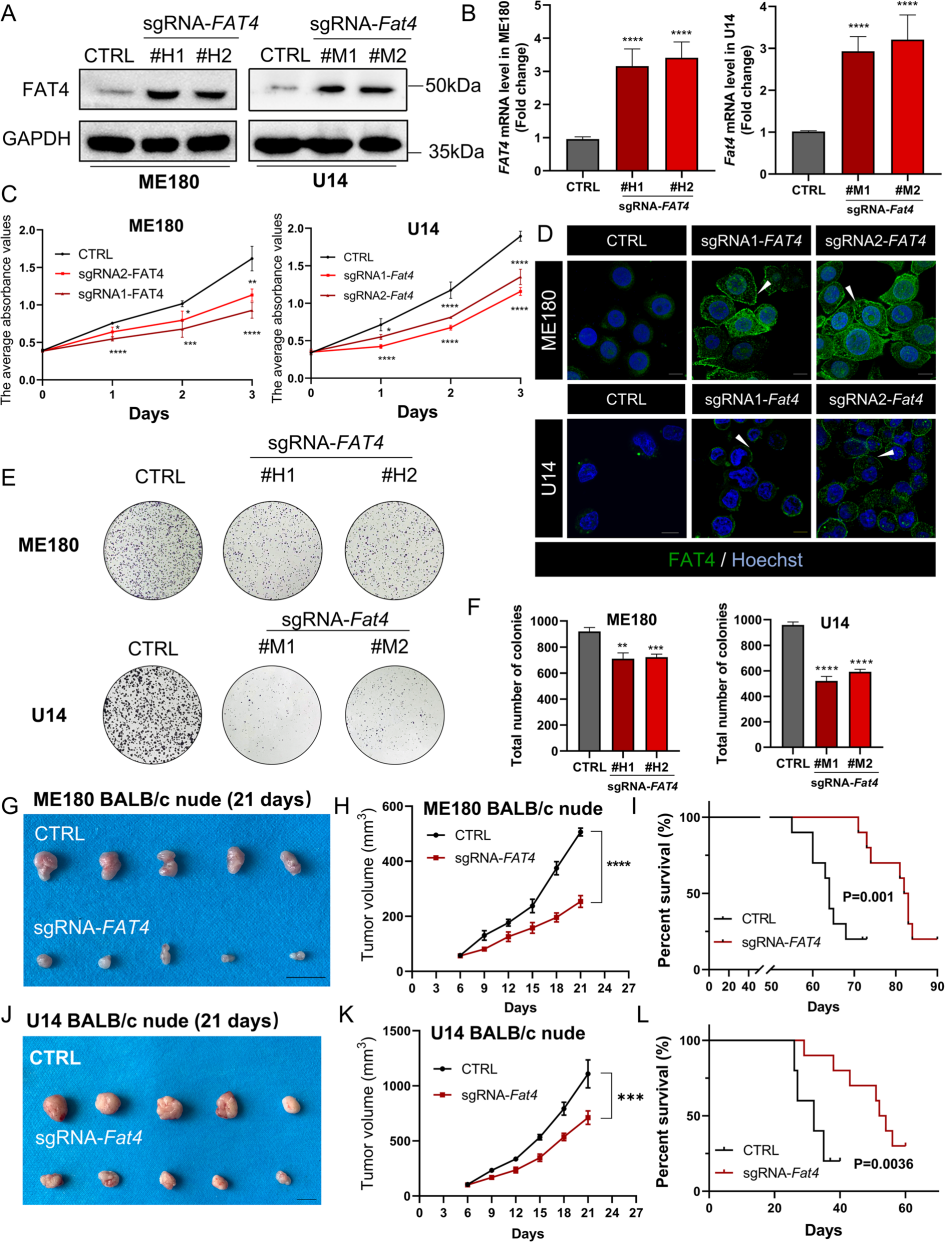
FAT4 overexpression inhibited cervical cancer proliferation both in vitro and in vivo. **A**&**B** Human-derived ME180 cell lines, and murine-derived U14 cell lines were transfected with sgRNA targeting the *FAT4/Fat4* or scrambled negative control (CTRL) sgRNA, and overexpression efficiency was analyzed by (**A**) immunoblotting and (**B**) RT-qPCR. **C** The CCK-8 assay was used to detect the proliferation of FAT4 overexpression cervical cancer cells and CTRL at the indicated time points. All error bars are expressed as mean ± SD, **P < 0.01, ***P < 0.001, and ****P < 0.0001. **D** Immunofluorescence detection of the subcellular localization of FAT4, note that cell membrane localization of FAT4 was significantly increased after overexpression (white arrow). **E**&**F **(**E**) Representative images and (**F**) the quantification results of colony formation assay in the indicated cervical cancer cells. Results are shown as mean ± SD. **G**-**I** Gross images of (**G**) negative control (CTRL) and sg*FAT4*-transfected ME180 xenografts in BALB/c nude mice, (**H**) tumor growth curves, and (**I**) Kaplan-Meier survival curves of these mice.** J**-**L** Gross images of (**J**) negative control and sg*Fat4*-transfected U14 xenografts in BALB/c nude mice, (**K**) tumor growth curves, and (**L**) Kaplan-Meier survival curves of these mice

Previous studies have shown that FAT4 inhibits tumor cell proliferation, but its role in cervical cancer is unknown [31–33]. FAT4 expression was detected in six cervical cancer cell lines using immunofluorescence and immunoblotting, with FAT4 expression being lower in ME180, C33A, and U14 cells and higher in SiHa, Caski, and Hela cells (Fig. 1H), but cell membrane localization of FAT4 was not observed in any of the above cell lines (Fig. 1I). Since the molecular weight of FAT4 full-length protein is 543 kDa, we used the deficient Cas9-synergistic activation mediator (dCas9-SAM) technology to transfect ME180 cervical cancer cell lines with short guide RNA (sgRNA) targeting the *FAT4* promoter region and endogenously promote FAT4 expression. As a control, dCas9 plasmid and a non-targeting sgRNA sequence (CTRL) were transfected. The same methods promote endogenous overexpression of FAT4 in U14 mouse cervical cancer cell lines (Fig. 2A and B). As FAT4 is a member of the cadherin superfamily, which regulates cell adhesion, immunofluorescence verified increased expression of FAT4 on cell membranes (Fig. 2D), especially at sites of cell-cell contact, which correlates with the regulation of intercellular contacts by the FAT protocadherin family. FAT4 overexpression in the ME180 and U14 cervical cell lines significantly suppressed cell proliferation and colony-forming efficiency, which was time-dependent (Fig. 2C, E and F).

For in vivo studies, we selected sg*FAT4* monoclone (#H1), and inoculated ME180 cervical cancer cells into immunodeficient (BALB/c nude) mice. When compared to the scrambled negative control group (CTRL, Cas9 plasmid and a non-targeting guide sequence), the tumor volume in the sg*FAT4*-transfected group was significantly reduced (Fig. 2G and H). Furthermore, compared to the CTRL group, the sg*FAT4* group had a longer survival time (Fig. 2I). We repeated the above experiments with the mouse cervical cancer cell line U14 and discovered that FAT4 overexpression could inhibit tumor growth (Fig. 2J and K) and prolong mouse survival (Fig. 2L). These data suggest that FAT4 overexpression inhibits tumor proliferation in immunodeficient mice.

### FAT4 increases CTL activity and change profiles of tumor-infiltrating lymphocytes in immune-competent mice

**Fig. 3:**
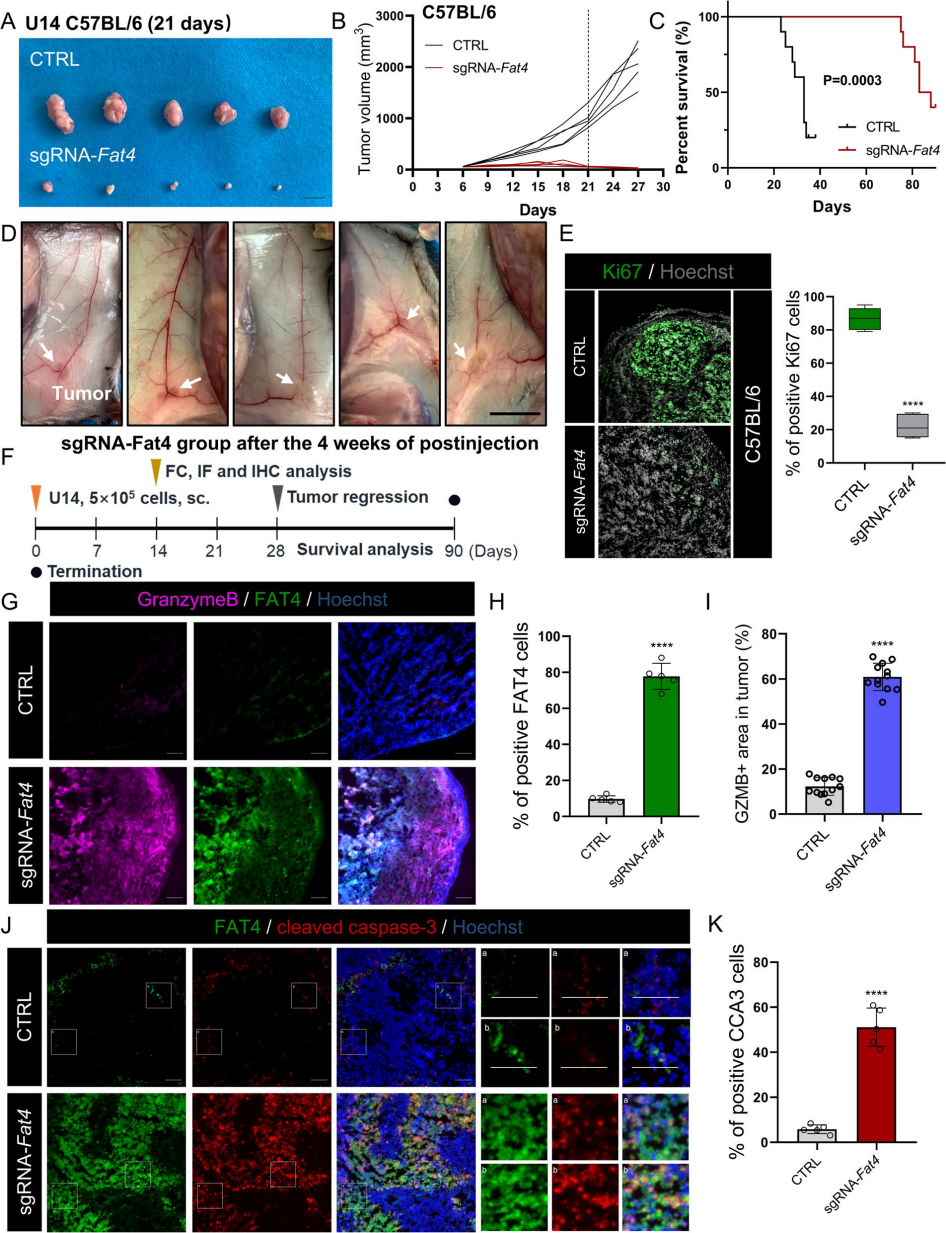
FAT4 overexpression promotes antitumor immunity in vivo.** A**-**C** Gross images of (**A**) negative control (CTRL) and sg*Fat4*-transfected U14 xenografts in C57BL/6 mice, (**B**) tumor growth curves, and (**C**) Kaplan-Meier survival curves of these mice. **D** Four weeks after inoculation of sgRNA-*Fat4* cells, regressed tumors were seen at the intersection of tumor neovascularization. **E** Ki67 (green) was visualized by IF, and nuclei were stained with Hoechst (dim gray) to calculate the Ki67 positive rate. **F** 5 × 10^5^ U14 cells were injected into immunocompetent C57BL/6 mice on day 0 and analyzed at the indicated time. sc, subcutaneous. FC, flow cytometry. IF, immunofluorescence. IHC, immunohistochemistry. **G**-**I** Co-stained of FAT4 and granzyme B (activity of T cell) in the U14 frozen tumor sections (**G**), nuclei were stained with Hoechst (blue) to calculate the FAT4 positive rate (**H**), and quantification of GZMB^+^ regions (4 tissue slides per tumor, 3 mice per group, *n* = 12). Results are presented as mean ± SD, Scale bar = 100 μm (**I**).** J**&**K** Co-stained of FAT4 and cleaved caspase-3 (CCA3) in the U14 frozen tumor sections (**J**), nuclei were stained with Hoechst (blue) to calculate the CCA3 positive rate (**K**). Scale bar = 100 μm

Intuitively, FAT4 overexpression in immunodeficient BALB/c nude mice reduced tumor size and prolonged survival (Fig. 2G-L). However, when sg*Fat4* cells were injected into syngeneic mice (C57BL/6), the differences in tumor growth and survival in the sg*Fat4* group were more pronounced than in the immunodeficient mice. When compared to the CTRL group, the tumor volume in the sg*Fat4* group was more obviously reduced around the third week after injection (Fig. 3A and B), and the survival time was prolonged in the sg*Fat4* group compared to the CTRL group (Fig. 3C). Furthermore, tumor growth was slow in syngeneic mice injected with sg*Fat4* U14 cells. Surprisingly, tumor regression from the injection site around the fourth week after injection, indicating that FAT4 overexpression activates antitumor immunity (Fig. 3D). Next, we used immunofluorescence to confirm that the proportion of Ki67-positive cells in the sg*Fat4* group was significantly lower than that in the CTRL group (Fig. 3E).

To investigate the mechanism of FAT4 overexpression activates antitumor immunity, we proceeded to construct subcutaneous graft tumors in immunocompetent C57BL/6 mice and collected tumors for further analysis before tumor regression (2 weeks after local injection, Fig. 3F). Cytotoxic T lymphocytes (CTLs) secrete high levels of perforin, granzyme B (GZMB), and interferon-γ (IFN-γ), which are the main effectors of antitumor immunity [34]. We used immunofluorescence to assess GZMB release and discovered that the sg*Fat4* group had considerably more GZMB^+^ region than the CTRL group (Fig. 3G-I). As GZMB targets caspase-3 upon entry into tumor cells and cleaved caspases to induce apoptosis [35], we compared the levels of cleaved caspase-3 (CCA3), where only a small number of cells underwent apoptosis in tumor tissues of CTRL mice, and strong aggregated apoptotic signals were widely observed in the sg*Fat4* mice (Fig. 3J and K).

**Fig. 4:**
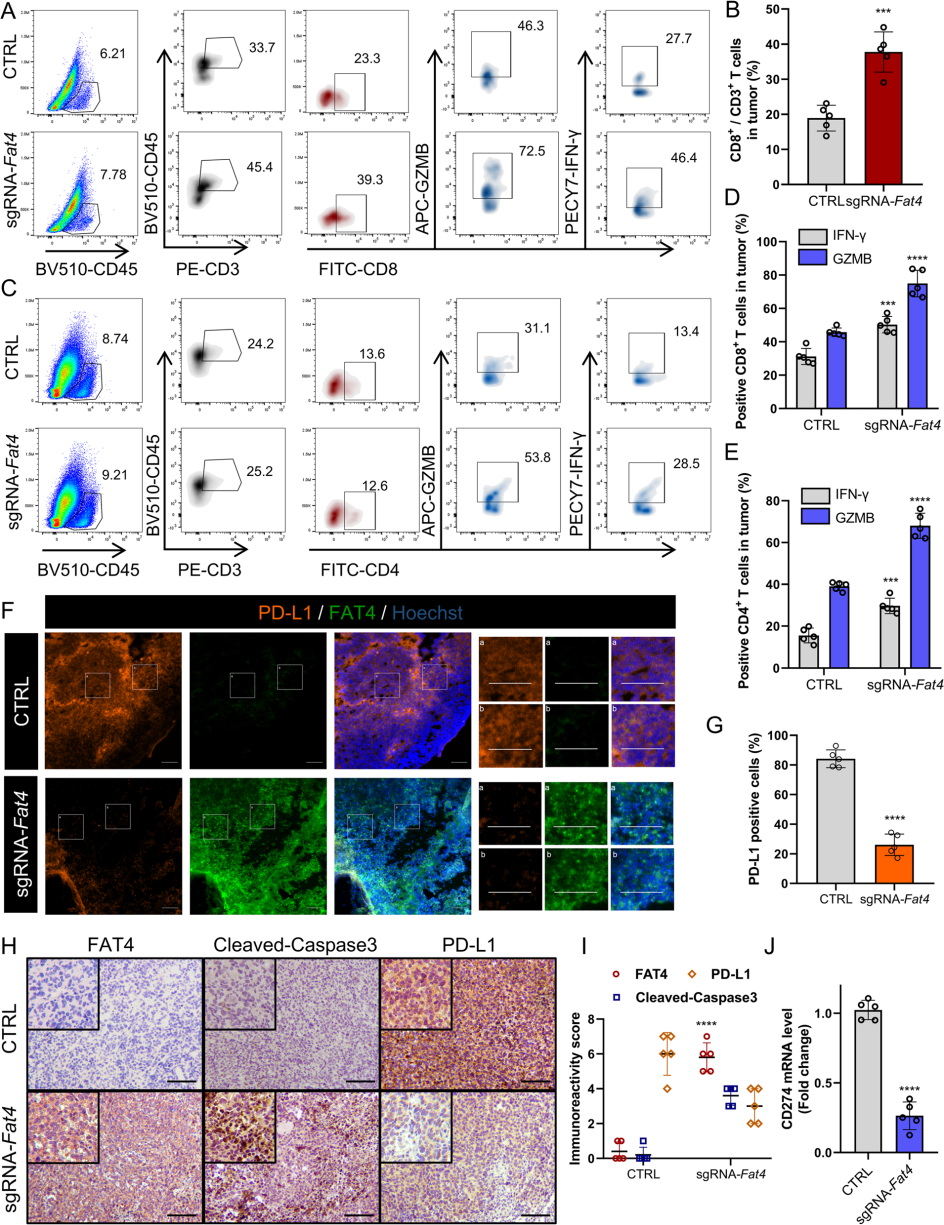
FAT4 overexpression activates CTL and inhibits PD-L1 expression in immunoreactive mice models. **A**&**D** Fluorescence-activated cell sorting (FACS) plots and quantification of CD8^+^ GZMB^+^ and CD8^+^ IFN-γ^+^ in CD3^+^ tumor infiltrating lymphocyte (TILs) derived from sg*Fat4* or CTRL group. All error bars are expressed as mean ± SD, ***P < 0.001 and ****P < 0.0001. **B** Fluorescence-activated cell sorting (FACS) quantification of CD8^+^ /CD3^+^ in CD45^+^ cells.** C**&**E** FACS plots and quantification of CD4^+^ GZMB^+^, CD4^+^ IFN-γ^+^ in CD3^+^ TILs derived from sg*Fat4* or CTRL group. **F**&**G** Co-stained of FAT4 and PD-L1 in the U14 frozen tumor sections (**F**), nuclei were stained with Hoechst (blue) to calculate the PD-L1 positive rate (**G**). Scale bar = 100 μm. **H**&**I** Immunohistochemical images (**H**) and immunoreactivity score (**I**) of FAT4, cleaved caspase-3, and PD-L1 expression in U14 C57BL/6 xenografts. All error bars are expressed as mean ± SD, **P < 0.01, ***P < 0.001 and ****P < 0.0001. **J.***Cd274* mRNA levels from the indicated U14 xenografts. Results are presented as mean ± SD, *n* = 5

Fluorescence-activated cell sorting (FACS) revealed a higher proportion of CD8^+^ (Fig. 4A and B) and a significantly higher CD8^+^ CTL activity (CD8^+^ GZMB^+^ and CD8^+^ IFN-γ^+^) population in the sg*Fat4* group (Fig. 4A and D). Similarly, we found that CD4^+^ CTL releasing GZMB and IFN-γ had considerably higher levels (Fig. 4C and E). In addition, the exhaustion of infiltrating CD4^+^ T cells (Fig. S1A and B) and CD8^+^ T cells (Fig. S1C and D) reflected by PD-1^+^ FACS staining was significantly reduced. Since cancer cells inhibit CTL activity through immune checkpoints, we continued to investigate whether FAT4 overexpression improved PD-L1-mediated immune escape. Immunofluorescence showed that in the sg*Fat4* group, the membrane localization of PD-L1 was significantly reduced (Fig. 4F and G). The above findings were also supported by immunohistochemistry (IHC), which revealed significantly higher levels of CCA3 protein and lower levels of PD-L1 protein in the sg*Fat4* group (Fig. 4H and I). And real-time quantitative PCR (RT-qPCR) confirmed that Cd274 mRNA levels were lower in the in the sgFat4 group (Fig. 4J). Reduced PD-L1 expression was associated with a significant increase in activated tumor-infiltrating CD8^+^ T cells. In this section, we demonstrate that FAT4 overexpression prevented tumor progression in immunodeficient mice, increased CTL activity, and even promoted tumor regression in C57BL/6 immunoreactive mice, suggesting that an intact immune system enhances the antitumor efficacy of FAT4 overexpression.

### FAT4 binds β-catenin and promotes its degradation in cervical cancer cells

**Fig. 5:**
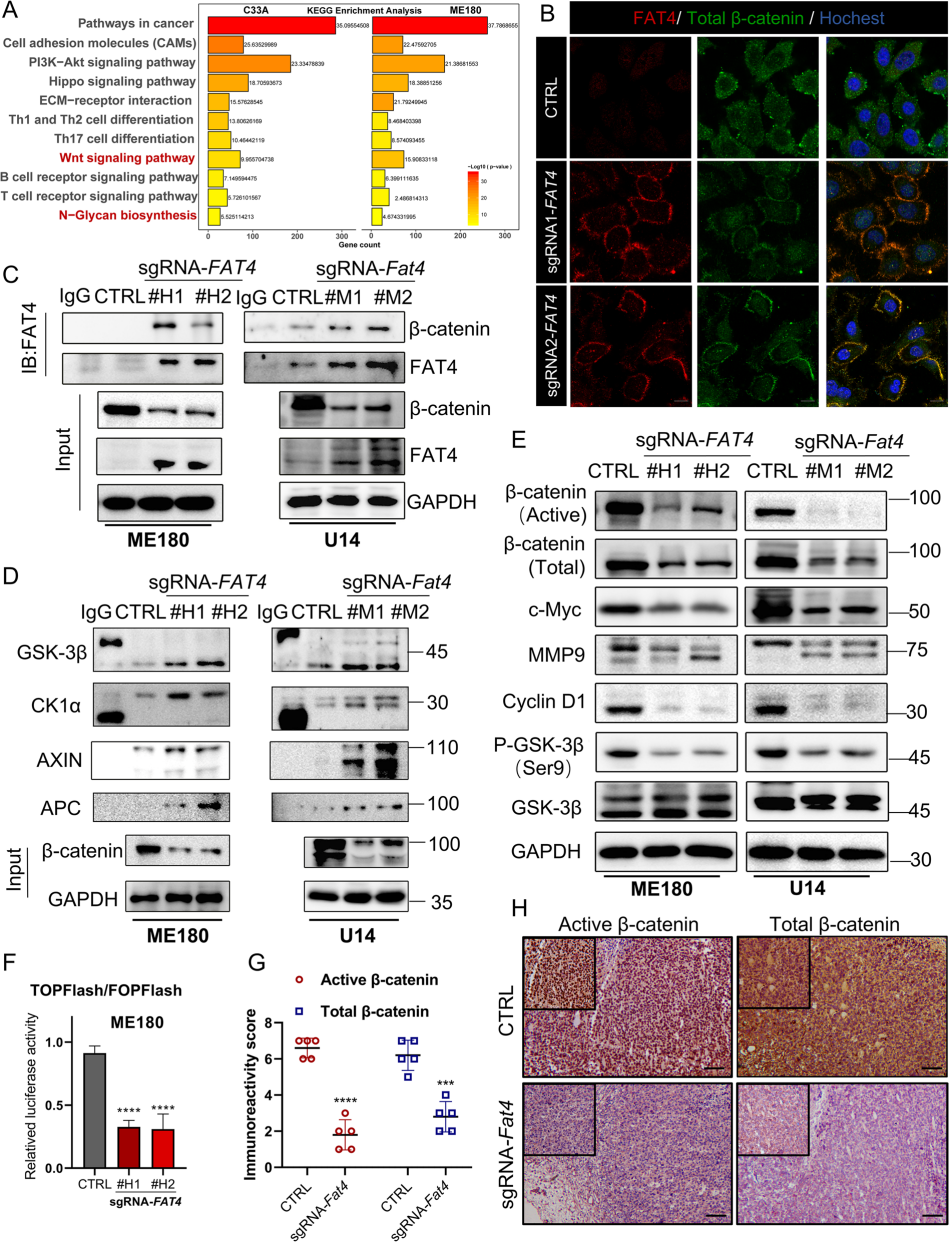
FAT4 overexpression inhibits the Wnt/β-catenin pathway in cervical cancer cells. **A** KEGG pathway enrichment analysis. Note that FAT4 overexpression is highly relevant for the Wnt signaling pathway, N-Glycan biosynthesis, and immune editing-related pathways and these findings are consistent in the C33A and ME180 cell lines. **B** Immunofluorescence staining for FAT4 (red) and total-β-catenin (green) in ME180 cells. In CTRL cells, the total-β-catenin runs throughout the cytoplasm, and sg*FAT4* is enriched in the cell membrane, with significant co-localization with FAT4. **C** The co-immunoprecipitation assay detects increased binding of overexpressed FAT4 to endogenous β-catenin. Immunoprecipitation with β-catenin antibody followed by immunoblotting with FAT4 antibody. **D** Interaction among the degradation complex (AXIN, GSK3β, CK1α, APC) and β-catenin in CTRL or FAT4 overexpression cells was determined by co-immunoprecipitation assay. Immunoprecipitation with β-catenin antibody followed by immunoblotting with AXIN, GSK3β, CK1α, and APC antibodies. **E** Immunoblotting was used to detect the expression of active-β-catenin, total-β-catenin, C-Myc, MMP9, and Cyclin D1, phospho-GSK-3β (Ser9) and total-GSK-3β in CTRL or FAT4 overexpression cells. **F** FAT4 inhibits β-catenin/TCF/TEF luciferase reporter activity. The TOPFlash/FOPFlash luciferase reporter assay was performed in CTRL or sg*FAT4* ME180 cells. pSV40-Renilla was used as an internal control. **G**&**H** Immunohistochemical images (**H**) and Immunoreactivity score (**G**) of active-β-catenin and total-β-catenin expression in U14 C57BL/6 xenografts. ***P < 0.001 and ****P < 0.0001

To identify the downstream signaling pathways of FAT4-mediated tumor suppression, we performed RNA sequencing on sg*FAT4* and non-targeting sgRNA sequence (CTRL) C33A and ME180 human cervical cancer cell lines. Based on KGEE enrichment analysis, differential alterations mainly encompassed lymphocyte activation, extracellular matrix-associated adhesion pathways, N-Glycan biosynthesis, and lymphocyte receptor pathways (Fig. 5A). There are indications that FAT4 homolog, protocadherin family FAT1 binds β-catenin in endothelial damage repair and human tumor cells [36, 37]. But whether this interaction exists between FAT4 and β-catenin is unclear. We first confirmed that FAT4 overexpression binds to β-catenin on the cell membrane in ME180 (Fig. 5B) and U14 cervical cancer cells (Fig. S2B). Endogenous FAT4 binding to β-catenin in ME180 and U14 cervical cancer cells was confirmed by co-immunoprecipitation (Fig. 5C). We then investigated whether FAT4 affects β-catenin subcellular localization, and observed that active β-catenin (non-phospho β-catenin, Ser45) in the CTRL group accumulates in the nucleus, and total β-catenin is detected throughout the cytosol with a reduced signal at the membrane (yellow arrows), whereas the nuclear localization of active β-catenin in the sg*FAT4* group was reduced, as well as an increase in membrane-associated β-catenin (white arrows, Fig. S2C). These findings were confirmed in U14 cells (Fig. S2D), implying that FAT4 overexpression anchors β-catenin to the cell membrane and prevents it from shuttling to the nucleus to initiate transcription.

The degradation complex (AXIN, GSK3β, CK1α, APC) mediates the phosphorylation of β-catenin in the cytoplasm, promoting its ubiquitination and subsequent proteasomal degradation [12, 38]. By immunoprecipitation, we found that the binding of the degradation complex to β-catenin was significantly enhanced in the FAT4 overexpression group compared to the CTRL groups (Fig. 5D), which directly led to ubiquitination-dependent degradation of β-catenin (Fig. S2A). Analogously, the expression of active β-catenin was significantly reduced, which is essential for β-catenin to mediate transcriptional activity through canonical Wnt signaling. Correspondingly, total β-catenin, phosphorylation-dependent GSK3β stabilization, and Wnt target genes MMP9, C-Myc, and cyclin D1 expression were significantly suppressed (Fig. 5E). Since the nuclear localization of β-catenin was significantly reduced, we assessed the transcriptional activity of β-catenin using TOPFlash/FOPFlash luciferase reporter assay [29], and β­catenin–mediated transcription was significantly decreased in the sg*FAT4* group (Fig. 5F). These findings were also confirmed in C57BL/6 immunoreactive mouse transplant tumors, where immunohistochemical staining showed a significant reduction in active β-catenin-positive signals in the nucleus, along with reduced staining for total β-catenin (Fig. 5G and H). These data suggest that FAT4 acts as a suppressor of Wnt/β-catenin signaling, “trapping” β-catenin at the cell membrane and promoting its degradation.

### FAT4 overexpression inhibits PD-L1 expression and cell membrane localization

The Wnt/β-catenin pathway is aberrantly activated to promote an immunosuppressive tumor microenvironment, while promoting immune checkpoint PD-L1 expression and cell membrane localization, causing T cells to become terminally exhausted and lose critical functions [39]. Specifically, β-catenin shuttles to the nucleus to promote transcription of CD274 and the N-glycosyltransferase STT3, which promotes PD-L1 glycosylation and ER-Golgi transport and maturation [13, 14]. The Edu assay confirmed that FAT4-induced proliferation inhibition could be partially reversed by PD-L1-WT plasmid transfection (Fig. S3B and C).

**Fig. 6:**
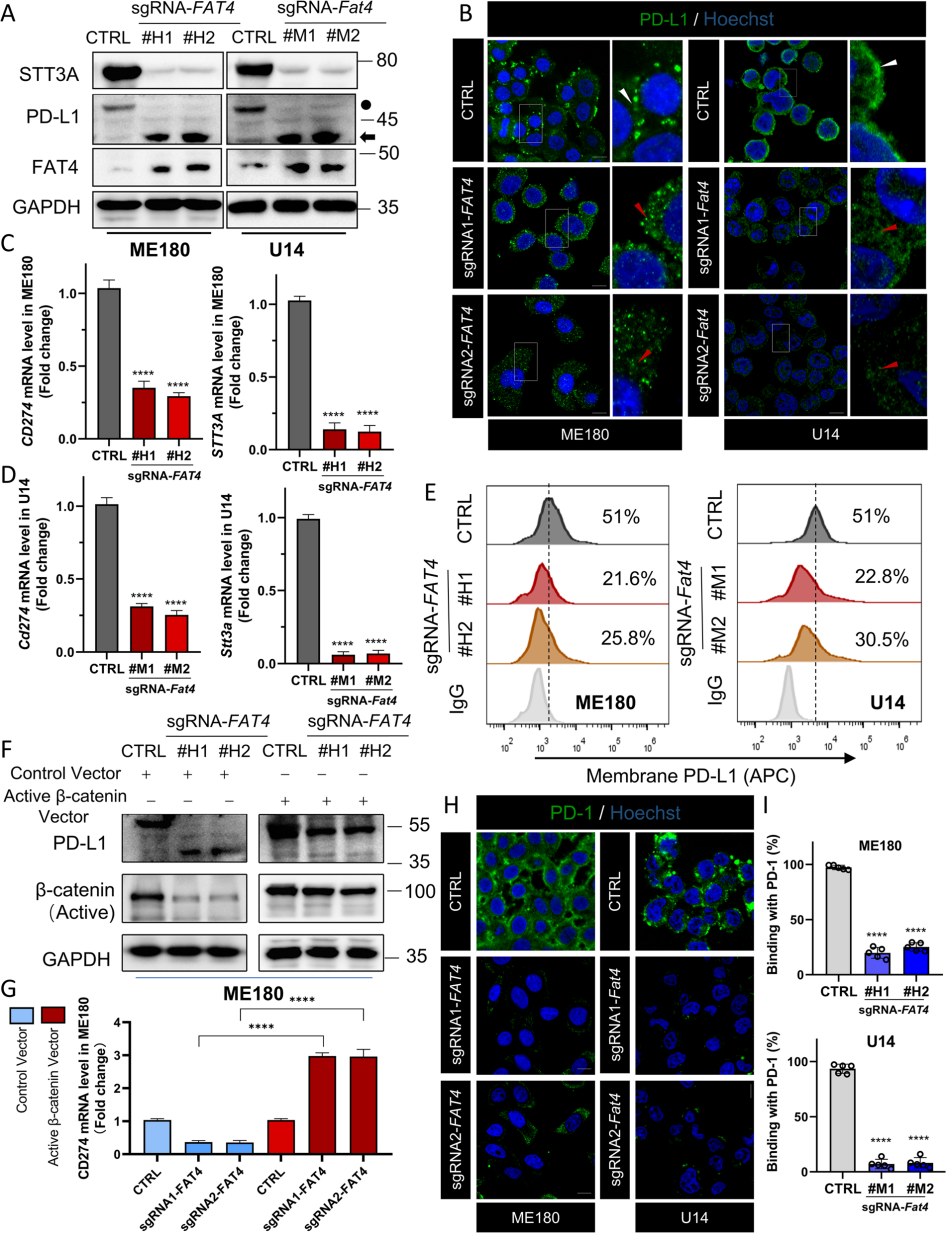
FAT4 overexpression inhibits PD-L1 expression and cell membrane localization. **A** Immunoblotting was used to detect the expression of PD-L1 and STT3A in CTRL or FAT4 overexpression cells. **B** Immunofluorescence staining for PD-L1 expression in ME180 and U14 cells. CTRL is located mainly on the cell membrane (white arrow). In FAT4 overexpression cells, FAT4 was detected throughout the cytoplasm with the reduced signal at the membrane (red arrows). Data represent mean ± SD. Scale bar = 10 μm. **C** RT-qPCR analysis of human CD274 and STT3A mRNA in sg*FAT4* and CTRL ME180 Cells. **D** RT-qPCR analysis of mouse Cd274 and Stt3a mRNA in sg*Fat4* and CTRL U14 Cells. **E** Flow cytometric analysis of PD-L1^+^ membrane expression in ME180 and U14 cells.** F**&**G** ME180 cells were transfected with control vector or Active β-catenin vector for 48 h. Immunoblot analysis (**F**) and RT-qPCR analysis (**G**) were performed. **H**&**I **(**H**) Representative images and (**I**) quantitation of green-fluorescent labeling recombinant human PD-1 Fc protein or recombinant mouse PD-1 Fc protein on CTRL or FAT4 overexpression cells. Scale bar = 10 μm. All error bars are expressed as mean ± SD, ***P < 0.001 and ****P < 0.0001

Recent research has revealed that PD-L1 is highly glycosylated on the membrane of tumor cells, and N-glycosylation of PD-L1, with a molecular weight of about 45 kDa on immunoblotting, which is crucial for PD-L1 protein stabilization by blocking PD-L1 from ubiquitin/proteasome-mediated destruction [24]. We found FAT4 overexpression decreased N-glycosylated PD-L1 (45 kDa, Black dot) while increasing non-glycosylated PD-L1 (33 kDa, Black arrow, Fig. 6A). Based on the findings in vivo, we equally found that FAT4 overexpression significantly decreased the expression of STT3A and PD-L1 at both protein and mRNA levels (Fig. 6A, C and D).

Since the active forms of PD-L1 are located on the cell membrane, we sought to demonstrate how FAT4 affected PD-L1 subcellular localization. Immunofluorescence confirmed that the CTRL PD-L1 was predominantly located on the cell membrane (Fig. 6B, white arrow), in contrast, sg*FAT4* PD-L1 signaling was detected throughout the cytoplasm (Fig. 6B, red arrow), with reduced signaling on the membrane. Flow cytometry revealed that FAT4 overexpression drastically reduced PD-L1 cell membrane expression (Fig. 6E). We further investigated that FAT4 overexpression reduces PD-L1 expression in a β-catenin-dependent manner in vitro. Expression of constitutively active β-catenin mutants (Active β-catenin) [28] increased total PD-L1 protein expression and CD274 mRNA expression (Fig. 6F and G) and promoted PD-L1 glycosylation maturation (45 kDa, Fig. 6F). Notably, Active β-catenin expression abolished the inhibitory effect of FAT4 overexpression on CD274 mRNA (Fig. 6G). These findings suggest that FAT4-induced β-catenin inactivation results in downregulation of PD-L1 in tumor cells. Functionally, we demonstrated that reduced PD-L1 expression on the membrane resulted in a significant reduction in PD-1 binding (Fig. 6H and I). And FAT4 overexpression in U14 cells enhanced T cell-mediated tumor cell killing (Fig. S3F and G). Collectively, these results suggest that FAT4 overexpression reduces PD-L1 levels in cancer cells in a β-catenin-dependent manner.

### FAT4 overexpression induces aberrant PD-L1 glycosylation and prevents its ER-to-Golgi translocation

**Fig. 7:**
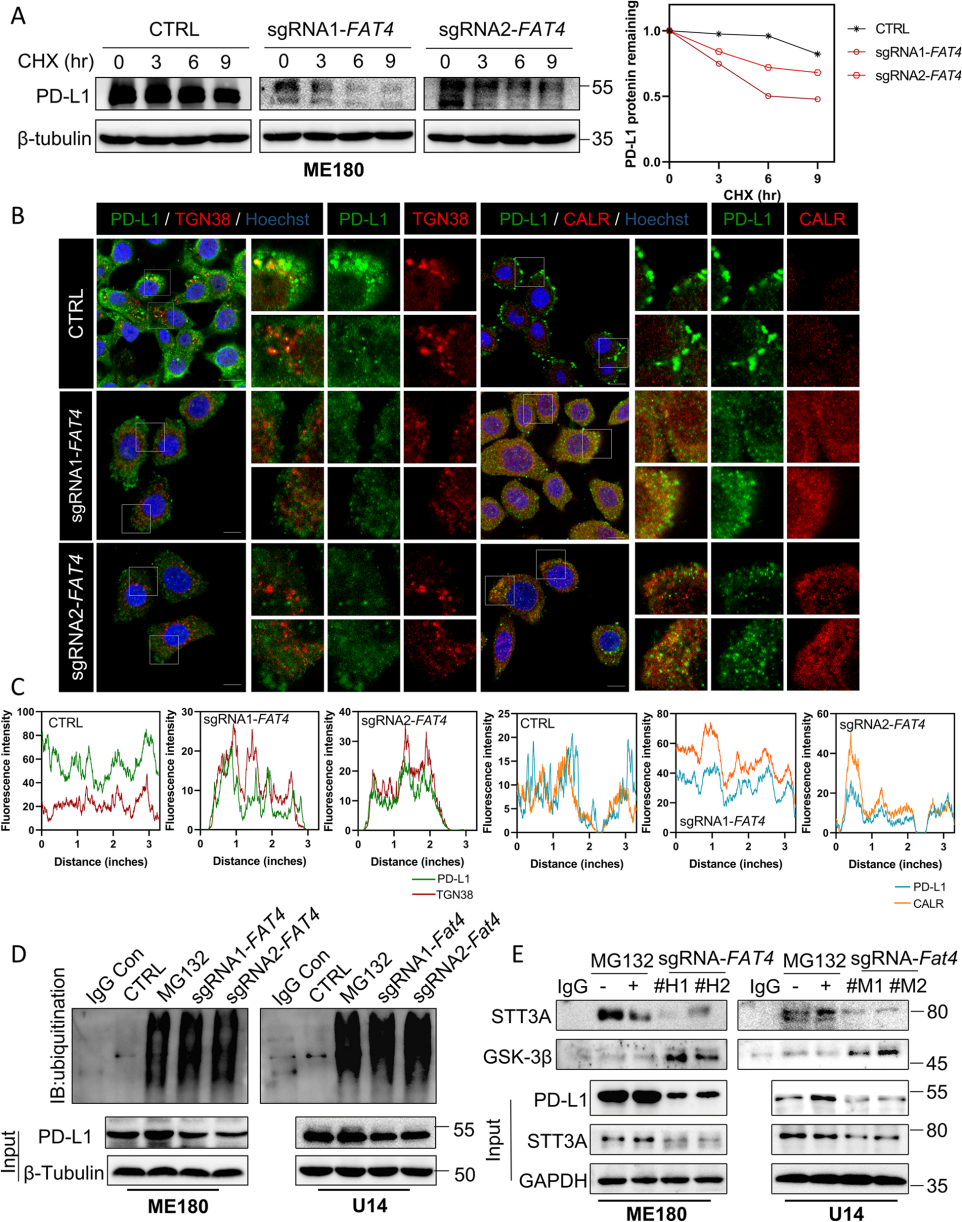
FAT4 overexpression induces abnormal PD-L1 glycosylation and prevents its ER-to-Golgi translocation. **A** Half-life analysis of PD-L1 in CTRL or sg*FAT4* cells were treated with 50 µg/mL actinomycin for the indicated times. PD-L1 levels were semi-quantified using β-tubulin as a loading control. At time 0, the relative PD-L1 levels were set to 1. **B** Immunofluorescence analysis of CTRL or sg*FAT4* ME180 cells for (left) co-localization of endogenous PD-L1 and endoplasmic reticulum maker (TGN38), (right) co-localization of endogenous PD-L1 and Golgi maker (Calreguli, CALR), nuclei stained with Hoechst (blue). **C** Intensity profiles showing signals from two fluorescent channels. **D** Ubiquitination of PD-L1 protein in CTRL or FAT4 overexpression cells. Immunoprecipitation was performed with PD-L1 antibody followed by immunoblotting with ubiquitin antibody. MG-132 (5 μM; 24 h) treated CTRL cells as a positive control. **E** Interaction among GSK-3β, STT3A, and PD-L1 in CTRL or FAT4 overexpression cells was determined by co-immunoprecipitation assay. Immunoprecipitation with PD-L1 antibody followed by immunoblotting with GSK-3β and STT3A antibodies

The particular glycosylation of membrane glycoproteins in the endoplasmic reticulum (ER) lumen during intracellular transport is intimately related to Golgi trafficking [40]. Glycosylated PD-L1 increases protein stability, and the protein half-life is at least 4-fold longer than non-glycosylated PD-L1 [24]. Next, we performed half-life analysis and as expected, PD-L1 half-life was shortened in FAT4 overexpressing cells (Fig. 7A). Co-staining of PD-L1 with Golgi markers (TGN38) and ER markers (Calregulin, CALR) showed that CTRL but not sg*FAT4* PD-L1 co-localized with Golgi marker, and sg*FAT4* but not CTRL PD-L1 co-localized with the endoplasmic reticulum marker (Fig. 7B and C). These findings were confirmed in U14 cells (Fig. S3A and B), implying that FAT4 blocks the ER-Golgi transport of PD-L1 thereby inducing PD-L1 accumulation in the ER. The oligosaccharide transferase complex (OST) catalytic subunit STT3 is required for PD-L1 glycosylation and protein stabilization, and the binding of β-catenin to the transcription factor TCF7L2 is required for the induction of STT3A/B transcription [14]. Glycosylation stabilizes PD-L1 and protects PD-L1 from GSK3β-mediated 26S proteasome-dependent ubiquitination degradation [24]. As expected, and endogenous PD-L1 ubiquitination was significantly increased in the FAT4 overexpression cells (Fig. 7D). We discovered that FAT4 overexpression decreased STT3A-PD-L1 interaction while increasing GSK3β-PD-L1 interaction, indicating that FAT4 inhibits the initiation of PD-L1 glycosylation in a β-catenin-dependent manner (Fig. 7E). These data suggest that FAT4 causes non-glycosylated PD-L1 in ER to undergo GSK3β-mediated proteasomal-ubiquitination degradation.

## Discussion

The FAT cadherin family displays similar extracellular domain structures, consisting of single-pass transmembrane receptors with 32–34 cadherin repeat sequences. The four FAT proteins invertebrates have different intracellular cytoplasmic domains, which may reflect the specific function of each FAT cadherin member [1, 4]. FAT4, the vertebrate FAT protein with the most sequence similarity to Drosophila Fat; there have been a few studies looking into the relationship between FAT4 and human cancers, and it has been proposed that FAT4 inhibits tumorigenesis and progression in endometrial [33], gastric [32], and colorectal [31] cancers. However, to our knowledge, the mechanism of FAT4-mediated β-catenin expression and whether FAT4 is associated with immune responses have not been discussed. Here, we report that FAT4 activates antitumor immunity through β-catenin by promoting aberrant glycosylation and degradation of PD-L1 and inhibiting the Wnt/β-catenin signaling pathway. Our findings provide a novel mechanism to regulate the β-catenin/STT3/PD-L1 axis.

Furthermore, the Wnt signaling is essential in maintaining stem cellularity, driving invasiveness, and immune escape [12, 38]. Activation of the Wnt/β-catenin pathway in solid tumors prevents T cells spontaneous activation and infiltration into the tumor microenvironment, increasing resistance to immune checkpoint inhibitor (PD-1 and CTLA4) therapy. Blockade of β-catenin combined with immune checkpoint inhibitor has been demonstrated to promote complete tumor regression in homozygous mouse models of melanoma, breast cancer, neuroblastoma, and renal adenocarcinoma [41, 42]. Therefore, exploring the molecular mechanisms by which Wnt/β-catenin signaling affects immune surveillance in cervical cancer will provide new directions for cancer immunotherapy [43]. In this study, we found that FAT4 overexpression decreased β-catenin nuclear accumulation and increased FAT4 chelating β-catenin at the cell membrane, which is consistent with FAT4 ubiquitinating β-catenin and subsequently promoting its degradation, thereby preventing the Wnt signaling and tumor growth. Analysis of the TCGA database revealed that the FAT4 gene is mutated or deleted in multiple squamous cell carcinomas, suggesting that FAT4 mutations may be responsible for dysregulation of the Wnt signaling.

Previous studies have shown that PD-L1 levels can be regulated at both the transcriptional and post-translational modifications (PTMs) and that PD-L1 regulation is an important mechanism affecting the efficacy of PD-L1/PD-1 immunotherapy [27]. In particular, several key transcription factors, including STAT3 [44], c-Myc [45], HIF1/2α [46], and nuclear factor-κB (NF-κB) [47], as well as mitogen-activated protein kinase (MAPK) [48], epidermal growth factor receptor (EGFR) [49], AKT/mTOR pathway [50] and Wnt/β-catenin pathway [13] can also boost PD-L1 mRNA expression when they are mutated or hyperactivated. Aberrant alterations in PTMs directly affect PD-L1-mediated immune resistance, including STT3-dependent N-linked glycosylation of PD-L1, GSK3β-dependent polyubiquitination, and degradation of PD-L1, and B3GNT3-dependent palmitoylation of PD-L1. It was reported that the β-catenin/TCF/LEF complex binds to the CD274 gene promoter region to induce PD-L1 expression, and the β-catenin/TCF7L2 complex binds to the STT3 gene promoter region to induce STT3A/STT3B expression [13, 14]. Importantly, N-linked glycosylation of PD-L1 is required for its stabilization and translocation to the cell membrane, where it exerts immunological escape [24]. In the present study, we demonstrate that FAT4 overexpression inhibits PD-L1 transcription and further glycosylation modifications in a β-catenin-dependent manner. The RNA-seq data indicate that FAT4 regulates N-Glycan biosynthesis. Specifically, FAT4 can cause aberrant N-linked glycosylation and ER retention of PD-L1 via the ER-associated N-glycosyltransferase STT3A, preventing PD-L1 from translocating to the membrane. It has been reported that GSK3β binds to the C-terminal structural domain of non-glycosylated PD-L1, causing PD-L1 phosphorylation followed by polyubiquitination [24]. This is consistent with our study, where we confirmed that FAT4 was able to promote the interaction of GSK3β with PD-L1 and further ubiquitination-dependent degradation. Functionally, through T cell-mediated cancer cell killing assays and PD-L1/PD-1 binding assays, we demonstrated that FAT4 overexpression in tumor cells significantly activated CTL activity by downregulating PD-L1 levels. Thus, our study reveals the molecular mechanism of tumor PD-L1 regulation. Finally, we discovered a novel FAT4 protein expression profile in cervical cancer and showed that FAT4 regulates immune editing, suggesting that the FAT4/β-catenin/STT3/PD-L1 signaling axis could be a potential target for cervical cancer.

### Supplementary Information

**Additional file 1: Figure S1.** Related to Fig. 4. (A&B) (A) Fluorescence-activated cell sorting (FACS) plots and (B) quantification of CD4^+^ PD-1^+^ in CD3^+^ TILs derived from sg*Fat4* or CTRL group. (C&D) (C) Fluorescence-activated cell sorting (FACS) plots and (D) quantification of CD8^+^ PD-1^+^ in CD3^+^ TILs derived from sg*Fat4 *or CTRL group. All error bars are expressed as mean ± SD, ****P< 0.0001. **Figure S2.** Related to Fig. 5. (A) Ubiquitination of β-catenin protein in CTRL or FAT4 overexpression cells. Immunoprecipitation was performed with β-catenin antibody followed by immunoblotting with ubiquitin antibody. MG-132 (5μM; 24 h) treated CTRL cells as a positive control. (B) Immunofluorescence staining for FAT4 (red) and total-β-catenin (green) in U14 cells. In CTRL cells, the total-β-catenin runs throughout the cytoplasm, and sgFat4 is enriched in the cell membrane, with significant co-localization with FAT4. (C&D) Immunofluorescence staining for active-β-catenin and total-β-catenin in (C) ME180 and (D) U14 cells. FAT4 overexpression significantly inhibited the nuclear localization of active-β-catenin, total-β-catenin was detected throughout the cytoplasm (yellow arrow) in CTRL cells, and sg*FAT4*/sg*Fat4* was enriched for signaling signals at the cell membrane (white arrow). **Figure S3.** Related to Fig. 7 (A) Immunofluorescence analysis of CTRL or sg*Fat4* U14 cells for (left) co-localization of endogenous PD-L1 and endoplasmic reticulum maker (TGN38), (right) co-localization of endogenous PD-L1 and Golgi maker (Calreguli, CALR), nuclei stained with Hoechst (blue). (B&C) The Edu assay confirmed that PD-L1 overexpression in sg*FAT4* ME180 cells could partially rescue the FAT4-induced proliferation inhibition (***P＜0.0010). (D&E) Intensity profiles showing signals from two fluorescent channels in Figure A. (F&G) T cell-mediated cancer cell killing assay. U14 CTRL and sg*Fat4* cells were co-cultured with activated T cells for 48 h and stained with crystal violet. U14 to T cell ratio 1:3. The normalized ratio of live cells in each well is shown. ***P< 0.001 and ****P< 0.0001. **Supplementary Table 1.** Antibody list.

## Data Availability

For all data requests, please contact the corresponding author.

## Abbreviations

DVL: Dishevelled segment polarity protein
TCF/LEF: T-cell factor/lymphoid enhancer-binding factor
CTL: Cytotoxic T lymphocyte
PCP: Planar cell polarity
TME: Tumor microenvironment
EMT: Epithelial-mesenchymal transition
Treg: Regulatory T cells
ICB: Immune checkpoint blockade
PD-1: Programmed cell death 1
PD-L1: Programmed death ligand 1
ER: Endoplasmic reticulum
OST: Oligosaccharyltransferase complex
TCGA: The Cancer Genome Atlas
CESC: Cervical squamous cell carcinoma and endocervical adenocarcinoma
dCas9-SAM: Deficient Cas9-synergistic activation mediator
CC: Cervical cancer
PT: Pericarcinomatous tissue
IRS: Immunohistochemical score
CTRL: Non-targeting sgRNA sequence
GZMB: Granzyme B
IFN-γ: Interferon-γ
CCA3: Cleaved caspase 3
FACS: Fluorescence-activated cell sorting
RT-qPCR: Real-time quantitative PCR
