# Supplementary material for: FAT4 overexpression promotes antitumor immunity by regulating the β-catenin/STT3/PD-L1 axis in cervical cancer
Source: J Exp Clin Cancer Res. 2023 Sep 1;42:222. doi: 10.1186/s13046-023-02758-2 (PMC10472690; doi:10.1186/s13046-023-02758-2)
Supplement: Supplementary file 1 — Additional file 1: Figure S1. Related to Fig. 4. (A&B) (A) Fluorescence-activated cell sorting (FACS) plots and (B) quantification of CD4+ PD-1+ in CD3+ TILs derived from sgFat4 or CTRL group. (C&D) (C) Fluorescence-activated cell sorting (FACS) plots and (D) quantification of CD8+ PD-1+ in CD3+ TILs derived from sgFat4 or CTRL group. All error bars are expressed as mean ± SD, ****P< 0.0001. Figure S2. Related to Fig. 5. (A) Ubiquitination of β-catenin protein in CTRL or FAT4 overexpression cells. Immunoprecipitation was performed with β-catenin antibody followed by immunoblotting with ubiquitin antibody. MG-132 (5μM; 24 h) treated CTRL cells as a positive control. (B) Immunofluorescence staining for FAT4 (red) and total-β-catenin (green) in U14 cells. In CTRL cells, the total-β-catenin runs throughout the cytoplasm, and sgFat4 is enriched in the cell membrane, with significant co-localization with FAT4. (C&D) Immunofluorescence staining for active-β-catenin and total-β-catenin in (C) ME180 and (D) U14 cells. FAT4 overexpression significantly inhibited the nuclear localization of active-β-catenin, total-β-catenin was detected throughout the cytoplasm (yellow arrow) in CTRL cells, and sgFAT4/sgFat4 was enriched for signaling signals at the cell membrane (white arrow). Figure S3. Related to Fig. 7 (A) Immunofluorescence analysis of CTRL or sgFat4 U14 cells for (left) co-localization of endogenous PD-L1 and endoplasmic reticulum maker (TGN38), (right) co-localization of endogenous PD-L1 and Golgi maker (Calreguli, CALR), nuclei stained with Hoechst (blue). (B&C) The Edu assay confirmed that PD-L1 overexpression in sgFAT4 ME180 cells could partially rescue the FAT4-induced proliferation inhibition (***P＜0.0010). (D&E) Intensity profiles showing signals from two fluorescent channels in Figure A. (F&G) T cell-mediated cancer cell killing assay. U14 CTRL and sgFat4 cells were co-cultured with activated T cells for 48 h and stained with crystal violet. U14 to T cell ratio 1 [file 13046_2023_2758_MOESM1_ESM.docx]

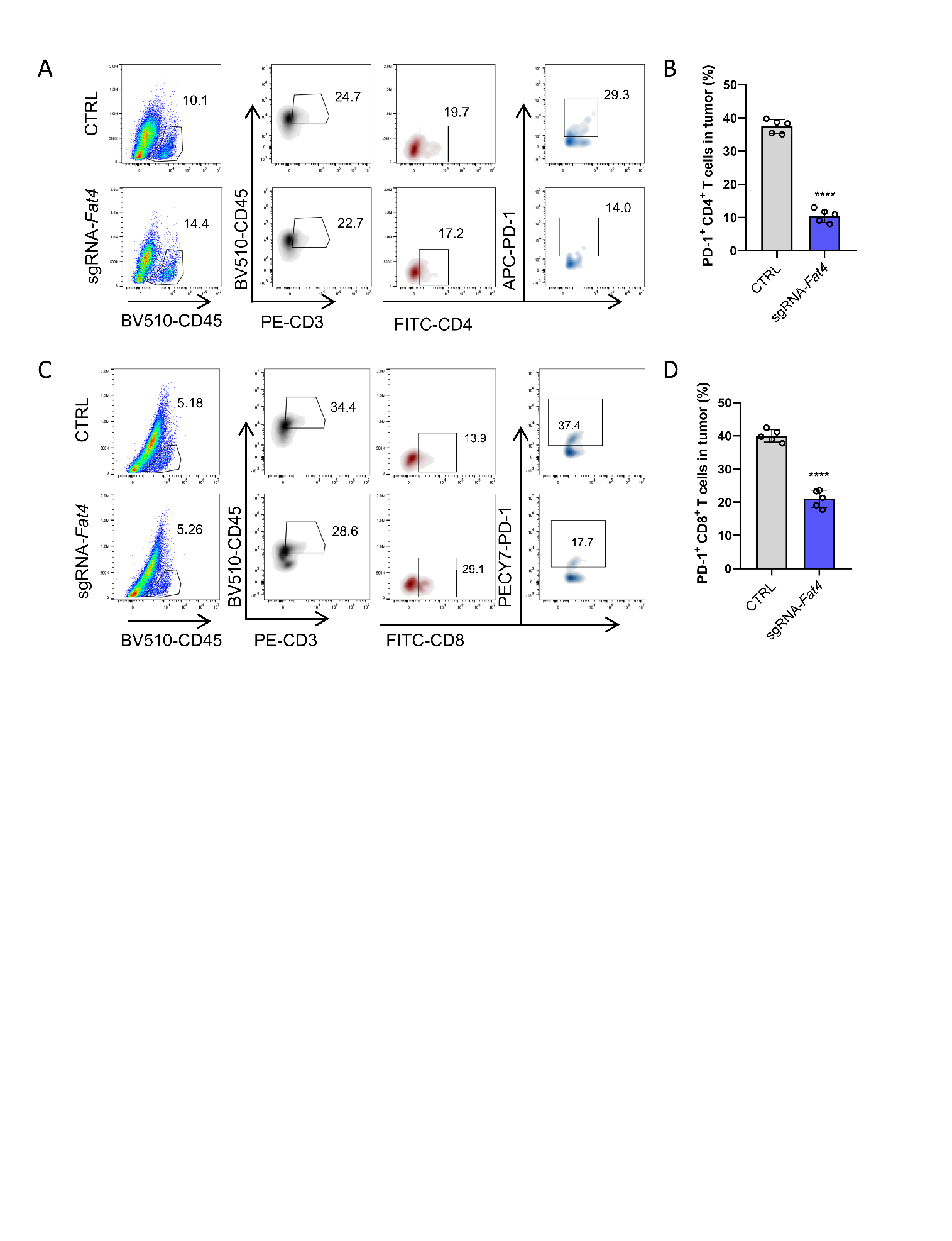
 **Additional file 1: Figure S1, related to Figure 4.** **(A&B)** (A) Fluorescence-activated cell sorting (FACS) plots and (B) quantification of CD4^+^ PD-1^+^ in CD3^+^ TILs derived from sg*Fat4* or CTRL group. **(C&D)** (C) Fluorescence-activated cell sorting (FACS) plots and (D) quantification of CD8^+^ PD-1^+^ in CD3^+^ TILs derived from sg*Fat4* or CTRL group. All error bars are expressed as mean ± SD, *****P*< 0.0001.


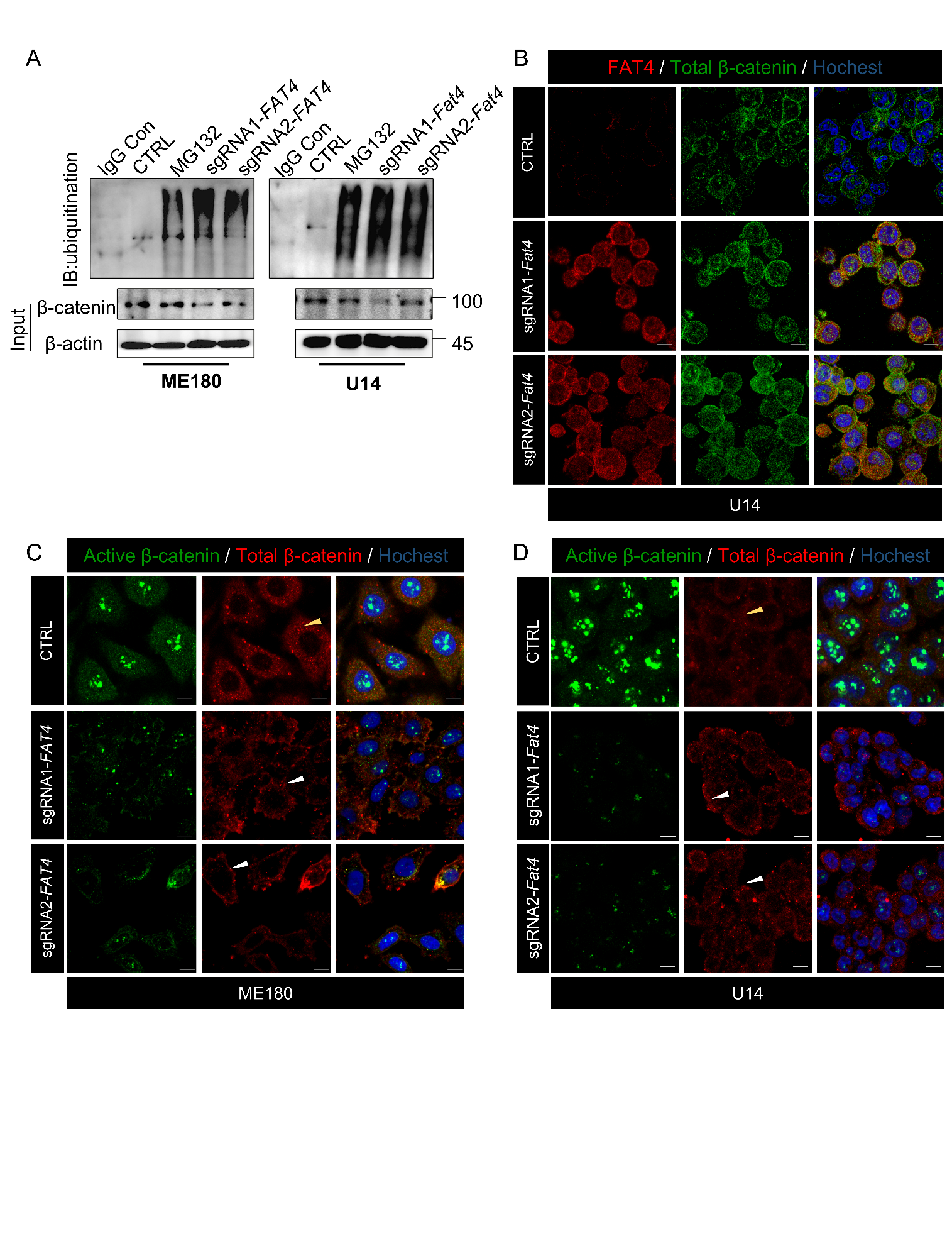
 **Additional file 1: Figure S2, related to Figure 5.** **(A)** Ubiquitination of β-catenin protein in CTRL or FAT4 overexpression cells. Immunoprecipitation was performed with β-catenin antibody followed by immunoblotting with ubiquitin antibody. MG-132 (5μM; 24 hours) treated CTRL cells as a positive control. **(B)** Immunofluorescence staining for FAT4 (red) and total-β-catenin (green) in U14 cells. In CTRL cells, the total-β-catenin runs throughout the cytoplasm, and sgFat4 is enriched in the cell membrane, with significant co-localization with FAT4. **(C&D)** Immunofluorescence staining for active-β-catenin and total-β-catenin in (C) ME180 and (D) U14 cells. FAT4 overexpression significantly inhibited the nuclear localization of active-β-catenin, total-β-catenin was detected throughout the cytoplasm (yellow arrow) in CTRL cells, and sg*FAT4*/sg*Fat4* was enriched for signaling signals at the cell membrane (white arrow).


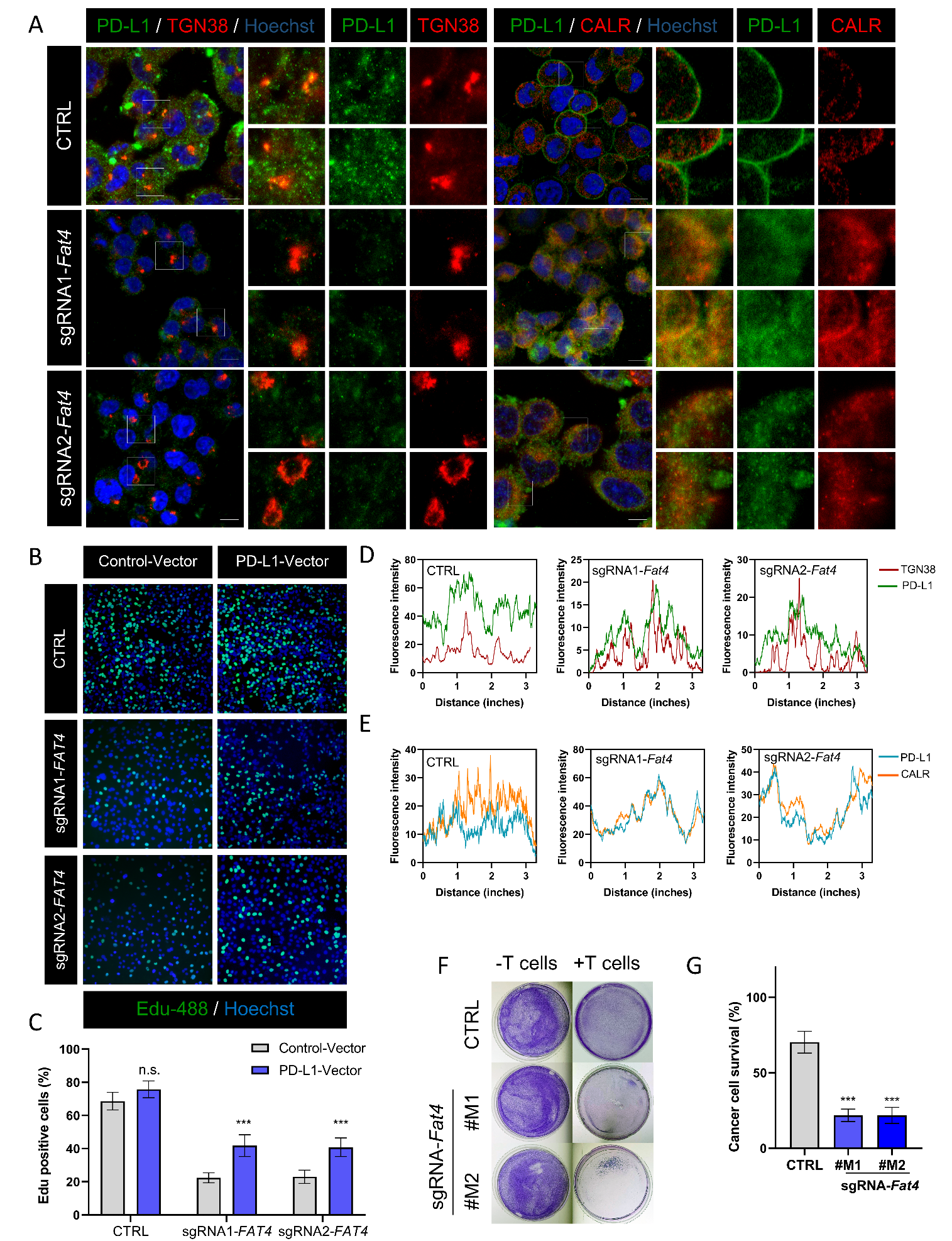


**Additional file 1: Figure S3 related to Figure 7 (A)** Immunofluorescence analysis of CTRL or sg*Fat4* U14 cells for (left) co-localization of endogenous PD-L1 and endoplasmic reticulum maker (TGN38), (right) co-localization of endogenous PD-L1 and Golgi maker (Calreguli, CALR), nuclei stained with Hoechst (blue). **(B&C)** The Edu assay confirmed that PD-L1 overexpression in sg*FAT4* ME180 cells could partially rescue the FAT4-induced proliferation inhibition (****P*＜0.0010). **(D&E)** Intensity profiles showing signals from two fluorescent channels in Figure A. **(F&G)** T cell-mediated cancer cell killing assay. U14 CTRL and sg*Fat4* cells were co-cultured with activated T cells for 48 h and stained with crystal violet. U14 to T cell ratio 1:3. The normalized ratio of live cells in each well is shown. ****P*< 0.001 and *****P*< 0.0001.

**Supplementary Table 1: Antibody list.**

| **Antibodies** | **Source** | **Host Species** | **Cat No.** | **Usage** | **Dilution** |
| --- | --- | --- | --- | --- | --- |
| CD45-Brilliant Violet 510™ | Biolegend | Rat | 103138 | FACS | 1:200 |
| CD3-PE | Biolegend | Rat | 100206 | FACS | 1:200 |
| CD4-FITC | Biolegend | Rat | 100406 | FACS | 1:200 |
| CD8-FITC | Biolegend | Rat | 100706 | FACS | 1:200 |
| Granzyme B-APC | Biolegend | Mouse | 372204 | FACS | 1:200 |
| PD-1-APC | Biolegend | Rat | 135209 | FACS | 1:200 |
| IFN-γ-PECY7 | Biolegend | Mouse | 502528 | FACS | 1:200 |
| PD-L1-APC | Biolegend | Mouse | 329708 | FACS | 1:200 |
| FAT4 | Abcam | Rabbit | ab130076 | WB/IF | 1:1000/1:100 |
| Non-phospho (Active) β-Catenin | Cell Signaling | Rabbit | 19807 | WB/IF/IHC | 1:1000/1:100/1:500 |
| PD-L1 (Extracellular Domain Specific) | Cell Signaling | Rabbit | 86744 | IF | 1:100 |
| Cleaved-Caspase-3 | Cell Signaling | Rabbit | 9661 | IF/IHC | 1:100/1:200 |
| MMP9 | Cell Signaling | Rabbit | 13667 | WB | 1:1000 |
| Phospho-GSK-3β (Ser9) | Cell Signaling | Rabbit | 9323 | WB | 1:1001 |
| FAT4 | Novus bio | Rabbit | NBP1-78381 | IHC | 1:150 |
| β-Catenin | Proteintech | Rabbit | 51067-2-AP | WB/IF/IHC | 1:1000/1:100/1:500 |
| PD-L1 | Cell Signaling | Rabbit | 13684 | WB | 1:200 |
| PD-L1/CD274 (C-Terminal) | Proteintech | Rabbit | 28076-1-AP | WB/IHC/IF | 1:500/1:500/1:100 |
| GSK-3β | Proteintech | Rabbit | 22104-1-AP | WB | 1:1000 |
| GAPDH | Proteintech | Rabbit | 10494-1-AP | WB | 1:10000 |
| β-Actin | Proteintech | Rabbit | 20536-1-AP | WB | 1:10000 |
| β-Tubulin | Proteintech | Rabbit | 20536-1-AP | WB | 1:2000 |
| ATP1A1 | Proteintech | Rabbit | 14418-1-AP | WB | 1:2000 |
| Ki67 | Proteintech | Rabbit | 27309-1-AP | IF | 1:150 |
| β-Catenin | Santa Cruz | Mouse | sc-7963 | IP | 2μg per 1ml cell lysate |
| PD-L1 | Santa Cruz | Mouse | sc-293425 | IP/IF | 2μg per 1ml cell lysate/1:100 |
| CD8α | Santa Cruz | Mouse | sc-7970 | IF | 1:100 |
| STT3A | Santa Cruz | Mouse | sc-390227 | WB | 1:500 |
| Ubiquitin | Santa Cruz | Mouse | sc-8017 | WB | 1:500 |
| Cyclin D1 | Santa Cruz | Mouse | sc-8396 | WB | 1:500 |
| CK1α | Santa Cruz | Mouse | sc-74583 | WB | 1:500 |
| APC | Santa Cruz | Mouse | sc-9998 | WB | 1:500 |
| AXIN | Santa Cruz | Mouse | sc-293190 | WB | 1:500 |
| c-Myc | Santa Cruz | Mouse | sc-40 | WB | 1:500 |
| Granzyme B | Santa Cruz | Mouse | sc-8022 | IF | 1:100 |
